# G Protein-Coupled Estrogen Receptor Agonist G-1 Inhibits Mantle Cell Lymphoma Growth in Preclinical Models

**DOI:** 10.3389/fonc.2021.668617

**Published:** 2021-06-15

**Authors:** Lixia Zhou, Tenghua Yu, Fei Yang, Jingjing Han, Bin Zuo, Lulu Huang, Xia Bai, Miao Jiang, Depei Wu, Suning Chen, Lijun Xia, Jia Ruan, Changgeng Ruan

**Affiliations:** ^1^ Jiangsu Institute of Hematology, National Clinical Research Center for Hematologic Diseases, NHC Key Laboratory of Thrombosis and Hemostasis, The First Affiliated Hospital of Soochow University, Suzhou, China; ^2^ Collaborative Innovation Center of Hematology, Soochow University, Suzhou, China; ^3^ Department of Breast Surgery, Jiangxi Cancer Hospital, Nanchang, China; ^4^ Cardiovascular Biology Research Program, Oklahoma Medical Research Foundation, Oklahoma City, OK, United States; ^5^ State Key Laboratory of Radiation Medicine and Protection, Soochow University, Suzhou, China; ^6^ Division of Hematology and Medical Oncology, Meyer Cancer Center, Weill Cornell Medicine, New York, NY, United States

**Keywords:** Mantle cell lymphoma, G protein-coupled estrogen receptor (GPER), G-1, cell proliferation, apoptosis, chemotherapy-free strategies

## Abstract

Mantle cell lymphoma (MCL) is an aggressive form of non-Hodgkin’s B-cell lymphoma with poor prognosis. Despite recent advances, resistance to therapy and relapse remain significant clinical problems. G-protein-coupled estrogen receptor (GPER)-mediated estrogenic rapid signaling is implicated in the development of many cancers. However, its role in MCL is unknown. Here we report that GPER activation with selective agonist G-1 induced cell cycle arrest, DNA damage, mitochondria membrane potential abnormality, and eventually apoptosis of MCL cell lines. We found that G-1 induced DNA damage and apoptosis of MCL cells by promoting the expression of nicotinamide adenine dinucleotide phosphate oxidase and the generation of reactive oxygen species. In addition, G-1 inhibited MCL cell proliferation by inactivation of NF-κB signaling and exhibited anti-tumor functions in MCL xenografted mice. Most significantly, G-1 showed synergistic effect with ibrutinib making it a potential candidate for chemotherapy-free therapies against MCL.

## Introduction

Mantle cell lymphoma (MCL) is a rare and aggressive form of B-cell lymphoma, characterized by the hallmark translocation (11;14) (q13; q32) that results in overexpression of cyclin D1 and cell proliferation (1). Additional genomic alterations, which are involved in cell cycle, DNA damage, signal transduction, and apoptosis (2, 3), are also found to contribute to MCL progression and resistance to conventional chemotherapy (4, 5). Recent advances in cytostatic drugs, including the development of immunomodulatory imide drugs such as lenalidomide and the Bruton’s tyrosine kinase (BTK) inhibitor such as ibrutinib, have established chemo-free regimes as a promising new direction of MCL therapy (5–7). However, several challenges remain. For example, primary and acquired resistance to ibrutinib is common, leading to poor prognosis (8, 9). Thus, there is a need to identify new targeted therapeutic options for MCL.

MCL has a high male/female ratio, which is up to 3.5:1, and male gender is considered an independent negative prognostic factor (10, 11). In addition to conventional estrogen receptors ER-α and ER-β, estrogens also mediate rapid signaling pathways *via* G-protein-coupled estrogen receptor (GPER), previously known as GPR30 (12). GPER is involved in non-genomic estrogenic signaling including calcium mobilization and generation of cyclic AMP, and stimulation of GPER activates matrix metalloproteinases, epidermal growth factor receptor (EGFR), ERK and PI3K pathways. Studies with GPER-deficient mice and GPER-selective agonist reveal that GPER exhibits an important function in various pathological processes including malignant diseases (13). GPER is expressed in various cancers, and studies in some female reproductive-related neoplasms suggest that higher GPER expression is associated with inferior prognosis and contributes to tumor development (14, 15). However, the opposite results were also observed (16–18). Study on GPER’s implication in MCL has just recently begun (19), and many questions remain unanswered.

G-1 is a GPER-selective agonist (20) and has been utilized to study GPER’s function. It has been shown that activation of GPER with G-1 represses proliferation and induces apoptosis in many cancers, such as ovarian cancer, colorectal cancer, breast cancer, and prostate cancer (21–24). In this study, we investigated the effects of G-1-induced GPER activation on MCL cells as well as on tumor growth in MCL-xenografted mice. In addition, we tested the potential combinative usage of G-1 and ibrutinib for MCL treatment in preclinical models.

## Materials and Methods

### Reagents and Antibodies

G-1 (B5455) and G-15 (B5469) were purchased from APExBIO (Texas, USA). G-36 (14397) was purchased from the Cayman Chemical Company (Michigan, USA). N-Acetyl-L-cysteine (NAC) (A9165) and 2′,7′-dichlorofluorescin diacetate (H_2_DCFDA) (D6883) were from MilliporeSigma (Massachusetts, USA). 2-Acetylphenothiazine (ML171) (S5304) and ibrutinib (S2680) were from Selleck (Texas, USA). FITC Annexin V Apoptosis Detection Kit with PI (640914) was purchased from BioLegend (California, USA). Hybrid-P protein hybrid nitrocellulose membranes (RPN303F) was from GE Amersham (Illinois, USA). BCA protein assay reagent kit (P0012), JC-1 (C2006), and propidium iodide (C1052) were from Beyotime (Shanghai, China). RevertAid First Strand cDNA Synthesis Kit (K1621), PowerUp™ SYBR™ Green Master Mix (A29742), and TurboFect Transfection Reagent (R0531) were from Thermo Fisher Scientific (Massachusetts, USA). Anti-mouse/rabbit immunohisto-chemical analysis kit (SP9000) was from ZSGB-Bio (Beijing, China). DAB Horseradish Peroxidase Color Development Kit (KGP1046) was from KeyGEN BioTECH (Jiangsu, China). siRNA targeting GPER and negative control siRNA were purchased from GenePharma (Shanghai, China). Antibodies against GPER (ab39742), CD20 (ab9475), NOX1 (ab55831), GAPDH (ab181602), anti-mouse IgG H&L (Alexa Fluor 594) (ab150116), and anti-Rabbit IgG H&L (Alexa Fluor 647) (ab150075) were from Abcam (Massachusetts, USA). Antibodies against H2A.X (7631), Phospho-H2A.X (Ser 139) (9718), Cleaved-Caspase-3 (9664), Cleaved-Caspase-9 (9505), Phospho- NF-κB p65 (Ser 536) (3033), NF-κB p65 (8242), anti-mouse IgG HRP-linked antibody (7076), and anti-rabbit IgG HRP-linked antibody (7074) were from Cell Signaling Technology (Massachusetts, USA).

### MCL Patients

MCL tumor tissues were collected from five male patients with an average age of 68.2 (age range, 62-74 years). These patients were admitted into The First Affiliated Hospital of Soochow University and were diagnosed with MCL according to the World Health Organization classification. Two control lymph nodes were from patients in the same hospital who received surgery due to gastric cancer and colon cancer, respectively. There was no metastasized cancer cell in any of the lymph nodes used in the present study, as certified by an experienced pathologist.

### Cell Lines

MCL cell lines (Jeko-1, Rec-1, Granta-519) were purchased from ATCC (Virginia, USA). Mino cell line was a kind gift from Dr. Jianhong Chu. Cells were cultured in RPMI 1640 medium containing 10% of fetal bovine serum (FBS) and 100 U/mL penicillin and 100 µg/mL streptomycin in a humidified incubator with 5% CO_2_ at 37°C. For assessment of apoptosis, cell cycle, mitochondrial membrane potential, and western blot, cells were cultured in 5×10^5^/mL and then were treated as indicated, respectively.

### Western Blot

To analyze protein expression, cultured cells were lysed using RIPA lysis buffer and the protein concentrations were determined by BCA protein assay reagent kit according to the manufacturer’s instructions. Lysate with total protein of 20 µg were applied to 10% or 12% SDS-PAGE, and separated proteins were transferred onto nitrocellulose membranes. After blots were blocked, the membranes were incubated with primary antibodies and then the corresponding secondary antibodies. Protein expression was visualized using Immobilon Western chemiluminescent HRP substrate.

### Immunofluorescence

To observe the cellular location of GPER, Mino cells were plated in polylysine-coated wells and fixed with 4% paraformaldehyde for 10 min, followed by permeation and blocking with 3% BSA, 3% donkey serum, and 0.3% Triton-100 for 1 h at room temperature. After blocking, the cells were incubated with primary antibodies against CD20 (1:50) and GPER (1:200) overnight at 4°C and then with fluorescence-conjugated secondary antibodies for 1 h at room temperature. DAPI Fluoromount-G was used for nuclear staining and sealing. Samples were analyzed with a confocal laser scanning microscope (TCSSP8, Leica, ×10).

To examine DNA damage, tumors from MCL-xenografted mice were fixed with 4% paraformaldehyde overnight and then dehydrated with 20% sucrose overnight followed by OCT embedding. Next, the tissues were cut into 5 µm slices and placed on glass slides. The slides were incubated with primary antibody against γ-H2A.X (1:400) overnight, then with the secondary antibody for 1 h. Sections were analyzed as described above.

### Quantitative Real-Time PCR

To evaluate the level of mRNA transcripts, total cellular RNA was isolated and reverse transcribed to cDNA with RevertAid First Strand cDNA Synthesis Kit according to kit instruction. ER-α, ER-β, and GPER mRNA expression were analyzed in duplicate by quantitative real-time PCR with PowerUp™ SYBR™ Green Master Mix using a 7500 Real Time PCR System (Thermo Fisher Scientific). The specific primers were “ATGGTCAGTGCCTTGTTGG-ATGC” and “GTCTGCCAGGTTGGTCAGTAAGC” for ER-α, “GCTGAACGCCGTGACCGATG” and “ACAGGAGCATCAGGAGGTTAGCC” for ER-β, “GGTGCTGGTCTTCTTCGTCTGC” and “AAGGC-GGCGAGGTTGACAATG” for GPER, and “GGTGCTGGTCTTCTTCGTCTGC” and “AAGGCGGCGAGGTTGACAATG” for GAPDH. The relative expression of these genes was calculated with the comparative cycle threshold (Ct) method (-ΔΔCt) by using GAPDH as endogenous control.

### Immunohistochemistry (IHC)

To test GPER expression in MCL patients, lymphoma tissues and lymph nodes from the patients were fixed and embedded in paraffin and then cut into 5 µm sections. After deparaffinization and dehydration, heat-induced antigen retrieval was performed. For incubating the samples with anti-GPER antibody and secondary antibody, the anti-mouse/-rabbit immunohistochemical analysis kit and DAB Horseradish Peroxidase Color Development Kit were used according to kit instructions. Finally, the slides were incubated with hematoxylin for 5 min for nuclear staining and were imaged by microscope (DM2000, Leica, 40×).

### Flow Cytometry

To study apoptosis, cultured cells were treated for 48 h or 72 h either with G-1 or G-36 dissolved in DMSO and other compounds as indicated, or with 0.2% DMSO alone as control. Cells were then assessed using FITC Annexin V Apoptosis Detection Kit with PI according to manufacturer’s protocol. For cell cycle assessment, cells were fixed with 70% ethanol for 12 h at -20°C followed by staining with PI. To determine mitochondrial membrane potential, G-1-treated cells were stained with JC-1 for 20 min at 37°C. To measure ROS levels, cultured cells were harvested after treatment with corresponding compounds for 24 h and then incubated with 5 µM of H_2_DCFDA (2′,7′-dichlorofluorescin diacetate) for 15 min at 37°C. After washing the cells with PBS three times, signal from fluorescent 2’,7’-dichlorofluorescein (DCF) was monitored. In all of the above experiments, labeled cells were differentially counted with CytoFLEX (FC500, Beckman Coulter) and data was analyzed with FlowJo software.

### Cell Viability Assay

Cell proliferation was determined using Cell Counting Kit-8. In short, 2×10^4^ cells suspended in 100 µL of 10% FBS-containing medium were seeded in 96-well plates and were then incubated with either G-1 alone, or ibrutinib alone, or a mixture of both, or G-36, all dissolved in DMSO or 0.2% DMSO alone as control, for 72 h or 120 h. After incubation with the CCK-8 solution for 4 h, the level of living cells was measured with SpectraMax M2 plate reader.

### siRNA Transfection

To knockdown GPER expression in MCL cells, 2 × 10^5^ Jeko-1 cells were cultured in 0.5 mL of 1640 medium with 10% of FBS. siRNAs were transfected into the cells using TurboFect Transfection Reagent according to manufacturer’s instructions. After 48 h, the cells were harvested for immunoblotting or were treated with G-1 for apoptotic analysis. Alternatively, transfected Jeko-1 cells were subjected for proliferation analysis at 48 h and 96 h and for apoptosis analysis at 48 h. SiRNAs were purchased from GenePharma (Shanghai, China). GPER siRNA, sense: 5’-3’ CCUGUGCUACUCCCUCAUUTT; anti-sense: 5’-3’AAUGAGGGAGUAGCACAGGTT. Control siRNA, sense: 5’-3’UUCUCCGAACGUGUCACGUTT; anti-sense: 5’-3’ ACGUGACACGUUCGGAGAATT.

### MCL Mouse Models

To generate MCL xenografted mice, 6×10^6^ Mino cells were subcutaneously injected into the right flank of 6-week-old male NOD/SCID mice. When the tumors were palpable after 2 weeks, the mice were randomized into two groups: vehicle control (n = 5) and G-1 treatment group (n = 5). G-1 was suspended in 5% DMSO and 95% normal saline. The mice in G-1 treatment group were intraperitoneally injected with G-1 (3 mg/kg/day), and the control group were intraperitoneally injected with the same volume of 5% DMSO and 95% normal saline. Tumor volumes were calculated every other day according to the formula: (the shortest diameter)^2^ × (the longest diameter) × 0.5. Thirteen days after G-1 administration, surgeries were performed on the mice under general anesthesia to remove tumors for further analysis.

### Statistical Analysis

The data are depicted as the mean ± SD of three independent experiments or the mean ± SEM of five cases. The difference between the two groups were analyzed by Student’s unpaired t test. Differences with P values less than 0.05 were considered statistically significant.

### Study Approval and Consent to Participate

Animal studies were conducted in accordance with the ethical guidelines approved by the Institutional Animal Care and Use Committee of Soochow University. Patient studies were approved by the Ethics Committee of the First Affiliated College of Soochow University. MCL Lymphoma tissues and lymph nodes of patients were acquired according to Declaration of Helsinki with written consent from them.

## Results

### Mantle Cell Lymphoma Cell Lines and Primary MCL Tumor Samples Express GPER

It has been demonstrated that MCL cell lines Jeko-1, Mino, Rec-1, and Granta-519 express GPER (19), To investigate the role of GPER, we first validated that GPER was expressed in the four cell lines under our experimental conditions. We detected GPER expression at both the protein level, as indicated by immunoblot analysis (**Supplemental Figure 1A**), and at the mRNA level by quantitative real-time PCR (**Supplemental Figure 1C**). Co-labeling Mino cells with anti-GPER and anti-CD20 antibodies showed predominant nuclear expression of GPER in these cells (**Supplemental Figure 1B**). Previous study has shown that lymphoma cells express ER-β, but not ER-α (25). We checked the mRNA levels of the two ER receptors in Jeko-1 and Mino cells, and our data were consistent with that finding (**Supplemental Figure 1C**). In addition, we examined tumor samples from lymph node biopsy of five MCL patients, and GPER expression was detected in four of them (**Supplemental Figure 1D**).

### GPER Antagonist G-36 Does not Exert Anti-Proliferative or Pro-Apoptotic Effects in Jeko-1 and Mino MCL Cells

GPER antagonist G-36 was shown to be able to reduce cell proliferation and induce apoptosis in MCL cell lines (19). To confirm this result, we treated Jeko-1 and Mino cells with G-36 (0.5 - 10 μM for 72 h). However, we did not observe the reported anti-proliferative or pro-apoptotic effects of G-36 in these MCL cells (**Supplementary Figure 2A, B**). We then validated this result with another GPER selective antagonist, G-15, and found that G-15 did not exert anti-proliferative or pro-apoptotic effects either even at a concentration of up to 10 µM for 72 h (data not shown). To further validate the effects of GPER inhibition on MCL cell growth, we knocked down GPER expression in Jeko-1 cells by transfecting the cells with a GPER specific siRNA. Comparing with cells transfected with control siRNA, GPER siRNA did not hinder cell growth at 48 h after transfection, and, in contrast, it even enhanced cell proliferation at 96 h (**Supplementary Figure 2C**). Furthermore, there was no apparent difference in cell apoptosis at 48 h between the two groups of cells (**Supplemental Figure 2D**). These data indicate that inhibition of GPER neither inhibits the proliferation nor promotes apoptosis of MCL cells in our experimental systems.

### GPER Agonist Induces Apoptosis of MCL Cells

**Figure 1:**
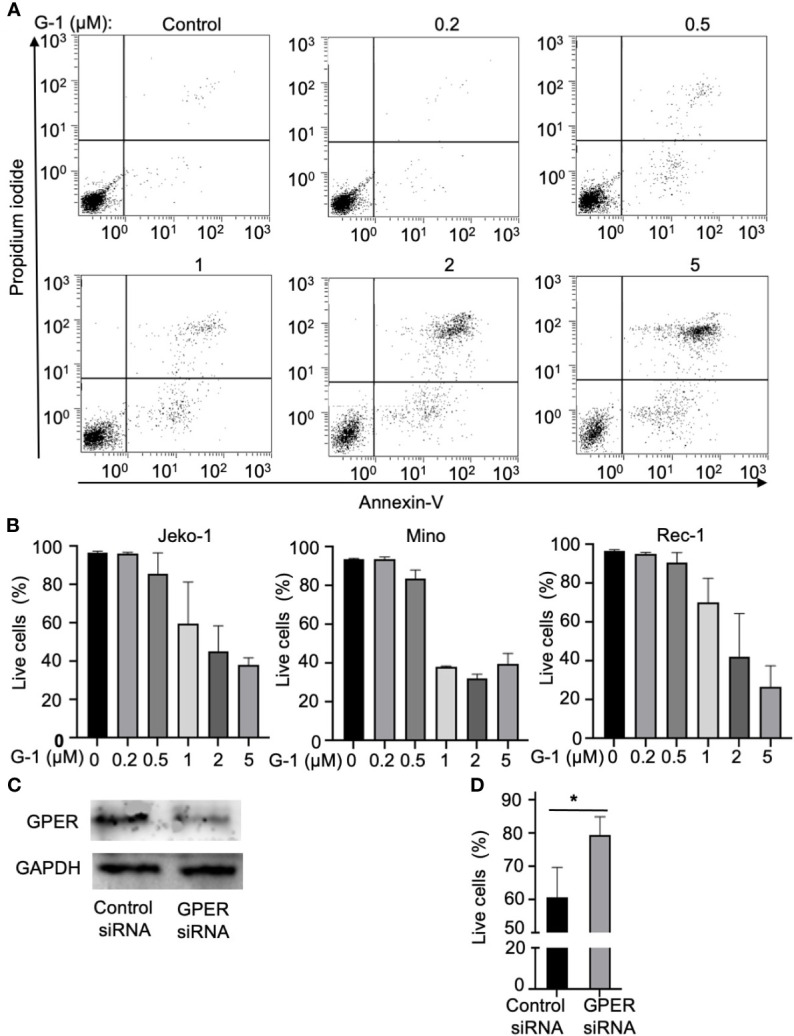
Treatment with GPER agonist G-1induces lethal effects in MCL cells. **(A)** Flow cytometry analysis of Jeko-1 cells after 48 h treatment of G-1(0 - 5 µM) followed by staining with Annexin-V/PI. Viable cells are annexin-V^-^/PI^-^. **(B)** Survival cell counts of Jeko-1, Mino, Rec-1 cells after 48 h treatment of G-1 followed by staining with Annexin-V/PI. Concentrations of G-1 is as indicated. **(C)** Immunoblot analysis of GPER expression in Jeko-1 cells transfected with control siRNA or siRNA for GPER. **(D)** Relative viable cell counts of above mentioned transfected Jeko-1 cells at 48 h after G-1 (1 µM) treatment. *P < 0.05.

GPER-selective agonist G-1 has been found to block tumor growths in various models (21–24). To determine the role of GPER in MCL, we treated multiple MCL cell lines, which included Jeko-1, Mino, Rec-1, and Granta-519 cell lines, with G-1 ranging from 0-5 µM. At 48 h after the treatment, cell death was observed in all cell lines except Granta-519 in a dose-dependent manner, as shown by a decrease in the number of viable cells (i.e. annexin-V^-^/PI^-^ cells) (**Figures 1A, B**). Granta-519 did not undergo obvious apoptosis even under the treatment with up to 5 µM of G-1 for 72 h (data not shown). MCL cell lines exhibit variable biology and clinical behavior influenced by their cytogenetic features and histopathologic origins (26). Granta-519 displays complex karyotypic and heterozygous deletion of TP53 (27). Different from the other three cell lines, Granta-519 carries Epstein Barr viral genomes and overexpresses Bcl-2, which has been characterized as an anti-apoptotic molecule through inhibiting caspase activation (28).

To rule out the nonspecific effect of G-1, GPER expression was suppressed using the siRNA against GPER (**Figure 1C**). Knockdown of GPER significantly abrogated the G-1-induced apoptosis in Jeko-1 (**Figure 1D**) which confirms that G-1 induces cell death through association with GPER. Mino and Rec-1 cells were highly susceptible to apoptosis caused by the transfection solution. Thus, these two cell lines were not used for this experiment.

### GPER Agonist Induces Cell Cycle Arrest and Caspases Activation

**Figure 2:**
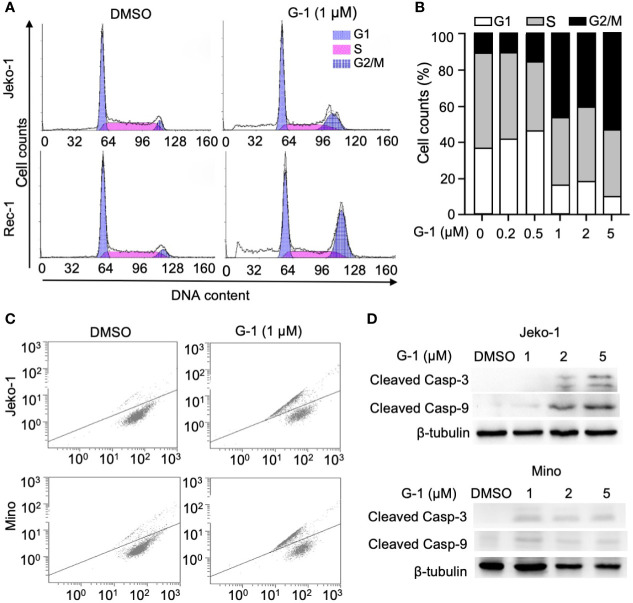
GPER agonist G-1 induces cell cycle G2 arrest and loss of mitochondrial membrane potential (MMP), and activates caspases in MCL cells. **(A, B)** Cell counts analyzed with flow cytometry of MCL cells at various cell cycle stages after 24 h of treatment with 1 µM **(A)** or 0-5 µM (**B**, Mino cells) of G-1 followed by PI staining. DMSO, treatment control; G1, S, G2/M, cell cycle stages. **(C)** Representative flow cytometry analysis of Jeko-1 and Mino cells after 48 h treatment with G-1 (1µM) followed by labeling with JC-1. **(D)** Immunoblot of protein levels in Jeko-1 and Mino cells after 48 h of incubation with G-1 in various concentrations. ß-tubulin, control of protein expression.

To explore the mechanisms of the apoptosis induced by G-1, the effects of G-1 on cell cycle was investigated. We treated Jeko-1 and Rec-1 cells with 1 µM of G-1 for 24 h and then analyzed the cell cycle stages *via* flow cytometry after propidium iodide staining (**Figure 2A**). Similar procedure was performed on Mino cells with an increasing amount (0-5 µM) of G-1 (**Figure 2B**). The results indicated that treatment with G-1 significantly increased the proportion of G2/M phase cells in a dose-dependent manner.

Mitochondria is at the heart of the intrinsic apoptosis pathway. Increased mitochondria membrane permeability leads to the release of cytochrome C which activates downstream caspases as well as reduces mitochondria membrane potential (loss of ΔΨm) (29). By monitoring the change of membrane-permeant fluorescent dye JC-1, we detected loss of ΔΨm in Jeko-1 and Mino cells upon G-1 treatment (**Figure 2C**). In line with this observation, the activation of caspase-3,9 was also observed in G-1-treated cells (**Figure 2D**).

### Treatment With G-1 Causes DNA Damage Through ROS Generation in MCL Cells

**Figure 3:**
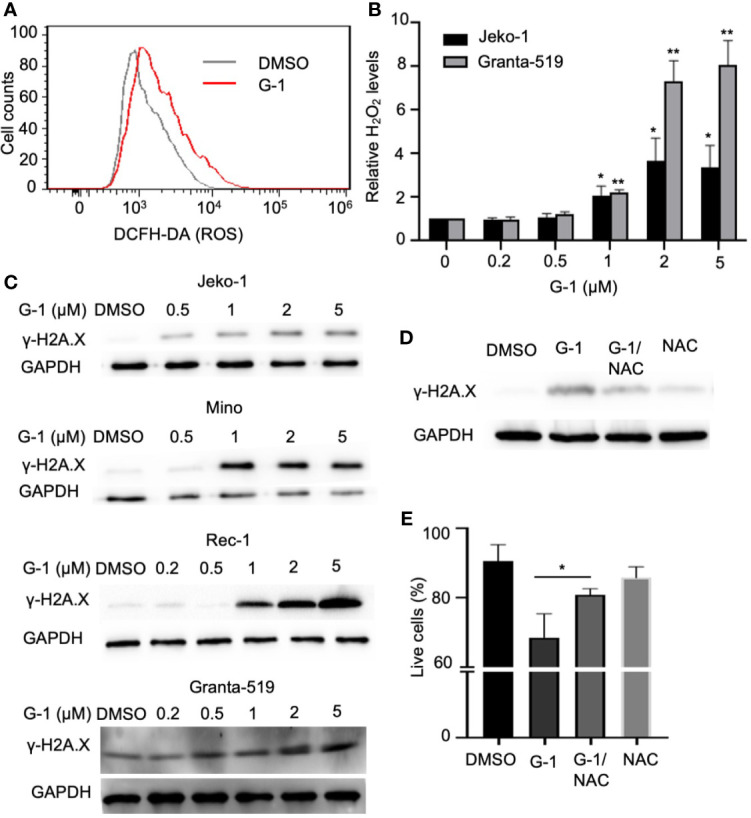
G-1 treatment induces ROS-dependent DNA damage in MCL cells. **(A)** Flow cytometry analysis of Mino cells treated with G-1 (1µM) or DMSO for 24 h followed by 15 min incubation with H_2_DCFDA. ROS was measured by detecting fluorescence of oxidized DCF. **(B)** Relative H_2_O_2_ level in G-1treated Jeko-1 and Granta-519 cells. Cells were incubated with 0 - 5 µM G-1 for 24 h and the value with 0 µM was used as 1, *P < 0.05, **P < 0.01. **(C)** Immunoblot analysis of phospho-H2A.X (γ-H2A.X) expression in G-1 (0 - 5 µM) treated MCL cells. ß-tublin, expression control. DMSO, treatment control. **(D)** Immunoblot analysis of phospho-H2A.X (γ-H2A.X) expression in 1 µM G-1 treated Jeko-1 cells for 24 h with or without pretreatment with 20 mM of NAC for 1 h. **(E)** Flow cytometry analysis of annexin-V^-^/PI^-^ Jeko-1 cells which were similarly treated as described in **(D)** for 48 h, *P < 0.05.

Cell cycle G2/M checkpoints can be triggered by DNA damage (30), and a high level of ROS induces DNA damage, which can result in mitochondrial dysfunction and even cell death (31). Since we observed cell cycle arrest, reduced mitochondria membrane potential as well as apoptosis in G-1 treated MCL cells, we speculated that G-1 could up-regulate ROS generation. Indeed, *via* monitoring the generation of oxidized DCF with flow cytometry, we found elevated levels of ROS in G-1-treated Mino cells compared with DMSO-treated cells (**Figure 3A**). In addition, increased levels of ROS were observed in G-1-treated Jeko-1 and Granta-519 cells in a concentration-dependent manner (**Figure 3B**). Furthermore, dose-dependent DNA damage by G-1 was indicated by the up-regulation of DNA double-strand break (DSB) maker, phospho-H2A.X (γ-H2A.X) (**Figure 3C**). Pretreatment of Jeko-1 cells with N-Acetyl-L-cysteine (NAC), an ROS scavenger, reduced G-1-induced ROS elevation (data not shown) and therefore mitigated the consequential DNA damage (**Figure 3D**) and cell death (**Figure 3E**), indicating that G-1 treatment leads to ROS generation and subsequent DNA damage, which in turn induces cell cycle arrest or apoptosis.

**Figure 4:**
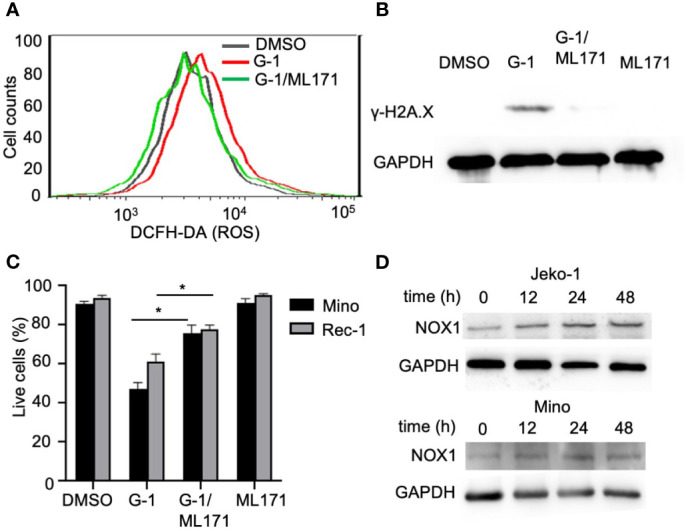
G-1 induced ROS and cytotoxicity is mediated by an NADPH oxidase. **(A)** Flow cytometry analysis of G-1 (1 µM)-treated Mino cells for 24 h with or without pretreatment of NOX1 inhibitor ML171 (50 µM) for 2 h. ROS levels were measured by monitoring the conversion of H_2_DCFDA to DCF. **(B)** Immunoblot analysis of γ-H2A.X expression in Mino cells treated as described in **(A)**. **(C)** Annexin-V^-^/PI^-^ cell counts of MCL cells treated as described in **(B)** for 48 h. *P < 0.05. **(D)** Immunoblot analysis of NOX1 expression in MCL cells treated with G-1 for various time.

NADPH oxidases (NOX) conduct various physiological and pathophysiological functions through regulating ROS generation and are enzymatic sources of ROS (32). Studies with GPER knockout mice have proven that GPER regulates NOX1 expression and function (33). We found that G-1 increased NOX1 expression in MCL cells (**Figure 4D**) and pretreatment with NOX1-specific inhibitor ML171 (34) significantly inhibited ROS production evoked by G-1 (**Figure 4A**). Importantly, it significantly inhibited DNA damage (**Figure 4B**) and cell death (**Figure 4C**). Therefore, it appears that G-1 induces ROS generation and cell death through activating NOX1.

### G-1 Down Regulates NF-κB Pathway and Exerts Synergistic Cytostatic Effects With Ibrutinib in MCL Cells

**Figure 5:**
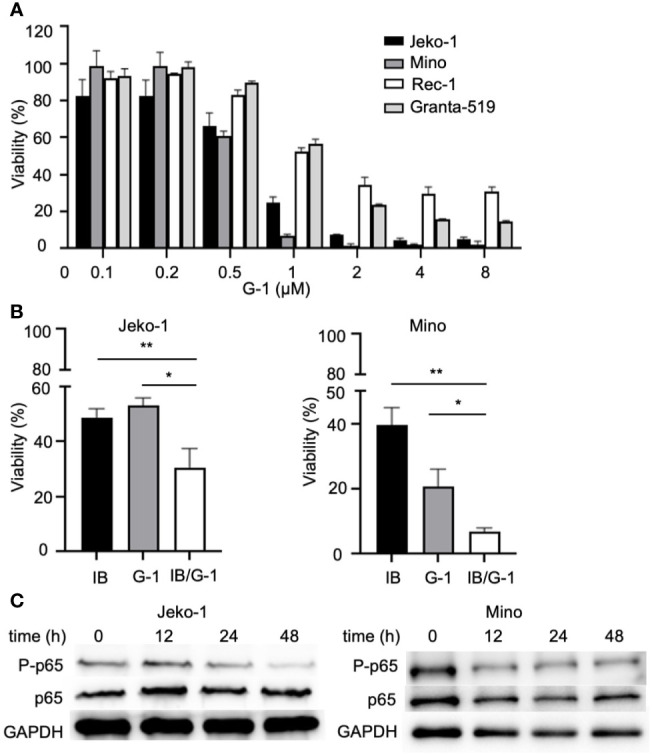
G-1 inhibits NF-κB activation and MCL cells proliferation. **(A)** Viable cell counts with cell counting kit-8 (CCK-8) of G-1 (0 - 8 µM; 72h)-induced MCL cells. Values of DMSO-treated cells were used as 100%. **(B)** Viable cell counts of variously treated Jeko-1 and Mino cells. IB, cells were treated with 4 µM of ibrutinib; G-1, cells were treated with 0.4 µM of G-1; IB/G-1, cells were treated with mixture of ibrutinib (4 µM) and G-1 (0.4 µM). *P < 0.05, **P < 0.01. **(C)** Immunoblot analysis of G-1 treated Jeko-1 and Mino cells with anti-P-p65 and anti-p65 antibodies. Durations of exposure to 1 µM of G-1 were indicated on top of the image.

To determine the effect of G-1 on MCL cell proliferation, we exposed MCL cells to various concentration of G-1 (0-8 µM) for 72 h. As shown in **Figure 5A**, G-1-treated groups have significantly fewer viable cells than the DMSO-treated group, demonstrating that G-1 inhibits the proliferation of MCL cells, including Granta-519, which is resistant to G-1-induced apoptosis.

NF-κB is well known to promote cell proliferation in cancer. It can be driven by upstream signal of B cell receptor and is involved in the pathogenesis of MCL (35). Studies showed that G-1 treatment could rapidly decrease the phosphorylation of p65 in triple-negative breast cancer cells, non-small cell lung cancer cells and colorectal cancer cells (21, 36, 37). To explore the association of NF-κB and GPER in MCL, we examined the expression of NF-κB in G-1 treated Jeko-1 and Mino cells. In agreement with the studies mentioned above, our experiments showed that after exposure to G-1 for different times (0-48 h), although the total p65 level remained stable, there was significant reduction of phosphorylated-p65 kinase (**Figure 5C**), indicating that G-1 inhibits MCL cell proliferation through down regulating NF-κB signaling pathway.

To define novel therapeutic strategies that may improve the anti-MCL function of BTK inhibitors, we assessed the combined effects of treatment with both ibrutinib and G-1 for 120 h in Jeko-1 and Mino cells. **Figure 5B** shows that co-treatment with ibrutinib and G-1 exerted synergistic inhibition of proliferation of both Jeko-1 and Mino cells. Furthermore, the synergy effect was observed in Granta-519 cells (data not shown).

### G-1 Shows Anti-MCL Activity in Mino-SCID Xenografted Mice

**Figure 6:**
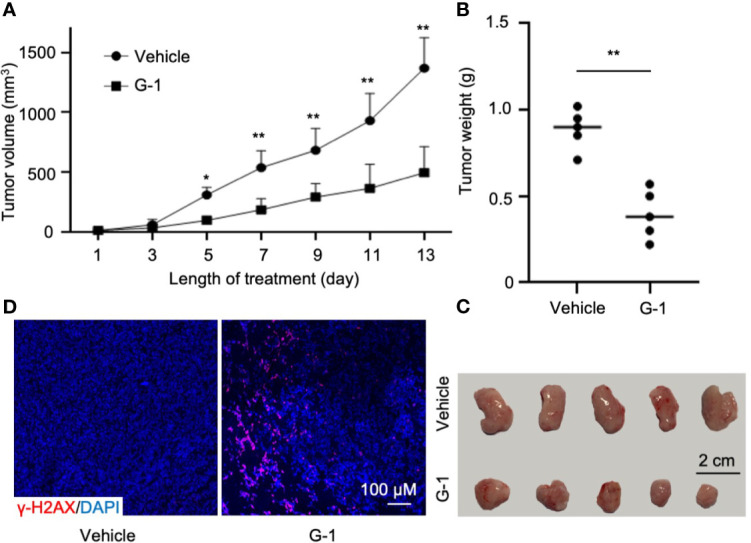
G-1 exerts anti-MCL activity in Mino-SCID xenografted mice. Mino cells (6 x 10^6^) were subcutaneously injected into SCID mice. Mice were treated with G-1 (3 mg/kg/d) or vehicle for 13 days (n=5 mice/group). **(A)** Tumor volume in xenografted mice at various times (days) post G-1 treatment. Vehicle, treatment control, *P < 0.05, **P < 0.01. **(B)** Tumor weights at day 13 after G-1 treatment, **P < 0.01. **(C)** Images of removed tumors. **(D)** Immunofluorescence staining with γ-H2A.X of tumor tissues from the xenografted mice.

Finally, we tested the *in vivo* effects of G-1 treatment on SCID mice xenografted with MCL cells. Following subcutaneous injection of 6×10^6^ Mino cells into the flanks of mice (n=5 per group), treatment with G-1 or control vehicle was commenced once tumor was visible. Thirteen days after G-1 administration, the mice were sacrificed and tumors were removed. Consistent with the observation with MCL cell lines, G-1 significantly inhibited tumor growth in all five mice (**Figures 6A–C**). Importantly, increased expression of γ-H2A.X was observed in tumors from all five mice in G-1 group compared with tumors from the control group (**Figure 6D**), an indication of elevated DNA damage in Mino-SCID xenografted mice induced by G-1.

## Discussion

In this study, we explored the effects of GPER agonist G-1 on MCL and investigated the mechanism of its action. We found that G-1 up-regulated NADPH oxidases expression in MCL cells that in turn led to DNA damage, cell cycle arrest and eventually apoptosis. G-1 also inactivated proliferation of MCL cells *via* down-regulating NF-κB pathway. In addition, G-1 significantly inhibited MCL tumor growth in xenografted mice. Furthermore, we found that G-1 exerted synergistic cytostatic effects with ibrutinib in MCL cells, suggesting that G-1 could be a potential candidate to combine with BTK inhibitor in chemo-free combinations for MCL.

Previous studies revealed that GPER deficiency in vascular smooth muscle cells led to the reduction of NOX1 mRNA and protein levels, and GPER antagonist G-36 selectively inhibits expression of NOX1 (33). Consistent with that, G-1 elevated the expression of NOX1 in MCL cell lines in our studies. NADPH oxidases play various physiological and pathophysiological roles through regulating ROS generation and oxidant stress can cause DNA damage (32). In our studies, we observed increased levels of ROS in G-1 treated cells. ROS in turn induced DNA DSB, a severe form of DNA damage, and this effect was abrogated by pre-treatment with ROS scavenger. Increased DNA damage was detected as well in tumor tissues from MCL mice. Cell cycle checkpoints, existing at G1/S and G2/M boundary, prevent cells from replication and entering mitosis in the presence of DNA damage to maintain the genome integrity (38). The G2 checkpoint can sense DNA DSB and trigger cell cycle arrest through DNA damage response pathway. Cells will re-enter cell cycle after the completion of DSB repair, or programmed cell death could be triggered (39). We detected cell cycle arrest and apoptosis in G-1 treated MCL cells. Observation of disruption of mitochondria membrane potential and elevated expression of cleaved caspases supports increased cell death. Our data show that G-1 induced DNA damage and apoptosis by regulating NOX1 and ROS generation, which is different from DNA damage caused by cytotoxic drugs. In addition, G-1 has been found effective in treating several cardiovascular and metabolic diseases *in vivo* (40, 41).

In addition to inducing apoptosis, we found that G-1 also inhibit MCL tumor cell growth by inactivating proliferation. It was shown that G-1 suppressed proliferation in several types of cancer cell lines by inhibiting NF-κB phosphorylation and cross-talk between GPER signaling and NF-κB signaling has been noted. For example, GPER activation reduces the secretion of inflammatory factors, such as IL-6 and TNF-α from monocyte/macrophages in mice, suggesting that GPER indirectly regulates NF-κB and reduces inflammation (42, 43). In human, GPER activation alleviates inflammation by interacting directly with the ER-α splice variant and the p65 subunit of NF-κB in primary monocytes (42). NF-κB pathway plays a crucial role in the proliferation and survival of lymphoid organs (44). Previous studies have documented that constitutive activation of NF-κB contributes to the pathogenesis of MCL (35), and ibrutinib has been approved in MCL patients for its function of inactivating NF-κB. However, MCL cells often become resistant to agents like ibrutinib due to alternatively activated NF-κB pathways. Thus, it is important to identify novel NF-κB – inhibiting therapeutics especially for relapsed or refractory mantle cell lymphoma. Our present studies demonstrated that in MCL cells, G-1 down-regulated the NF-κB pathway by decreasing the phosphorylation of p65 and G-1 and ibrutinib exhibited a synergistic effect on inhibiting proliferation in MCL cell lines, providing a possible treatment strategy for ibrutinib-resistant MCL patients.

G-1 significantly reduced tumor size of MCL xenografted mice. Some studies demonstrated that G-1 mediates inflammation and immunity *via* activating GPER in T cells and suppresses autoimmune disease (45, 46). Thus, whether the *in vivo* anti-lymphoma effect of G-1 is partly mediated by the tumor immune microenvironment of the MCL xenografted mouse needs further investigation.

Many cancer cell lines and primary tumors express GPER, including MCL.

Studies of GPER’s function in cancer progression and G-1’s effects on cancer cell lines or tumor tissues have shown conflicting results. For example, G-1 stimulates proliferation in primary breast cancer tissue and the GPER antagonist G-36 completely inhibits G-1-mediated proliferation (47); G-1 enhances ovarian cancer cell proliferation *via* EGFR and Akt signaling pathways (48). In contrast, G-1 induces cell cycle arrest and inhibits prostate cancer cell growth through activation of ERK and it substantially reduces tumor size of cancer cell xenografted mice (24); G-1 decreases adrenocortical carcinoma cell growth *in vivo* and *in vitro* by inducing apoptosis (49); In addition, G-1 triggers apoptosis *via* ROS/ERK and inhibits NF-κB in colorectal cancer cells and suppresses the *in vivo* progression of colorectal cancer (21). These data suggest that GPER plays diverse functions depending on cell types, underlying pathology, and tumor micro-environment. GPER is expressed in the early stages of immune cells including B cells (43). But related functional studies are very limited. Estrogen-GPER have been reported to regulate immune response of B cells by increasing IgG production in mice and antibody production in fish (50, 51). Mice lacking GPER do not exhibit obvious abnormality in B cells related immune development and function (41). Information regarding GPER in MCL is scarce. There is only one report (a letter to the editor) that reports an analysis of 157 MCL patients with no correlation of GPER expression with the Ki-67 proliferation index (19). It presented that GPER antagonist G-36 could inhibit proliferation of MCL cell lines, as assessed by the MTT assay, with IC50 of 1.4 - 8.9 µM (19). However, in our studies, G-36 and G-15 neither suppress MCL cell growth nor increase MCL cell apoptosis even at a concentration of up to 10 µM for 72 h. Furthermore, knocking down GPER expression in Jeko-1 did not restrain the cells from surviving. Rather, we found that agonist G-1 induced apoptosis and inhibited proliferation in MCL cells. Most significantly, in our *in vivo* study, G-1 obviously reduced the size of tumors from all MCL xenografted mice.

Our data support that G-1 could be a promising therapeutic candidate for MCL. First, GPER is expressed in majority of MCL patients (19) and any effective GPER-targeting therapy may benefit these patients. Secondly, molecular studies have identified the heterogeneous spectrum of somatic mutations in MCL, which results in the variable biology and clinical behavior of the disease (1). For example, TP53 and ATM had been reported as one of the most frequently mutated genes in MCL, and the patients with TP53 mutation have inferior prognosis (2). It is remarkable that low concentration of G-1 showed antitumoral activity, *via* pro-apoptosis and/or anti-proliferation functions, in MCL cell lines with either defective tumor suppressor gene (TP53 in Jeko-1 and Mino), DNA damage response gene (ATM in Granta-519), or cell-cycle checkpoint gene (p16 in Rec-1 and Granta-519) (52). These data imply that a broad range of MCL patients could be sensitive to G-1 treatment.

Recent advances in cancer drug discovery shines light on the development of chemo-free strategies in MCL management, which overcomes conventional chemotherapy-related toxicity. Several novel targeted therapies, including ibrutinib, have proven to be highly effective for relapsed/refractory MCL, while ongoing trials have demonstrated that depth and duration of response can be improved by combining novel agents with ibrutinib (5). However, the responses to ibrutinib in MCL patients appears not be lasting and relapse usually happens. Other than mutation in BTK gene itself, relative resistance to ibrutinib can be caused by non-genetic adaptive mechanisms leading to compensatory pro-survival pathway activation such as NF-κB (53). Our present study showed that GPER agonist G-1 inactivates NF-κB pathway and appears to have a synergistic effect with ibrutinib on inhibiting proliferation in MCL cells. Therefore, addition of G-1 might potentially improve the outcome of MCL patients who develop resistance to chemo-free therapies with ibrutinib.

## Data Availability Statement

The original contributions presented in the study are included in the article/**Supplementary Material**. Further inquiries can be directed to the corresponding author.

## Ethics Statement

The animal study was reviewed and approved by Institutional Animal Care and Use Committee of Soochow University.

## Author Contributions

LZ, DW, SC, LX, CR, and JR conceived and designed research. LZ, TY, FY, JH, BZ, LH, MJ, and XB performed the experiments and analyzed data. LZ, DW, LX, CR, and JR interpreted the data and wrote the manuscript. All authors contributed to the article and approved the submitted version.

## Funding

This study was supported by National Natural Science Foundation of China (81828001 and 81702641), the Priority Academic Program Development of Jiangsu Higher Education Institutions (PAPD), and the Jiangxi Science Fund for Distinguished Young Scholars (2018ACB21042).

## Conflict of Interest

The authors declare that the research was conducted in the absence of any commercial or financial relationships that could be construed as a potential conflict of interest.

## Abbrevations

MCL, Mantle cell lymphoma; GPER, G-protein-coupled estrogen receptor; BTK, Bruton’s tyrosine kinase; EGFR, epidermal growth factor receptor; ROS, reactive oxygen species; γ-H2A.X, phospho-H2A.X; NOX, NADPH oxidase; NF-κB, nuclear factor kappa-B; NAC, N-Acetyl-L-cysteine; H2DCFDA, 2′,7′-dichlorofluorescin diacetate; DSB, double-strand break.
