# Supplementary material for: G Protein-Coupled Estrogen Receptor Agonist G-1 Inhibits Mantle Cell Lymphoma Growth in Preclinical Models
Source: Front Oncol. 2021 Jun 15;11:668617. doi: 10.3389/fonc.2021.668617 (PMC8239310; doi:10.3389/fonc.2021.668617)

G protein-coupled estrogen receptor agonist G-1 inhibits mantle cell lymphoma growth in preclinical models

Lixia Zhou[1,2], Tenghua Yu[3], Fei Yang[1], Jingjing Han[1], Bin Zuo[1], Lulu Huang[1], Xia Bai[1,2,4,5] Miao Jiang[1], Depei Wu[1,2], Suning Chen[1,2], Lijun Xia[1,4,*], Jia Ruan[6,*], and Changgeng Ruan[1,2,4,5*]
[1]Jiangsu Institute of Hematology, National Clinical Research Center for Hematologic Diseases, NHC Key Laboratory of Thrombosis and Hemostasis, The First Affiliated Hospital of Soochow University, Suzhou, 215006, China. [2]Collaborative Innovation Center of Hematology, Soochow University, Suzhou, 215006, China. [3]Department of Breast Surgery, Jiangxi Cancer Hospital, Nanchang, 330029, China. [4]Cardiovascular Biology Research Program, Oklahoma Medical Research Foundation, Oklahoma City, Oklahoma, USA. [5]State Key Laboratory of Radiation Medicine and Protection, Soochow University, Suzhou, 215123, China. [6]Division of Hematology and Medical Oncology, Meyer Cancer Center, Weill Cornell Medicine, New York, NY, USA
*Co-senior authors

Raw data set: main raw data corresponding to each figure as labeled

Figure 1B

JEKO-1

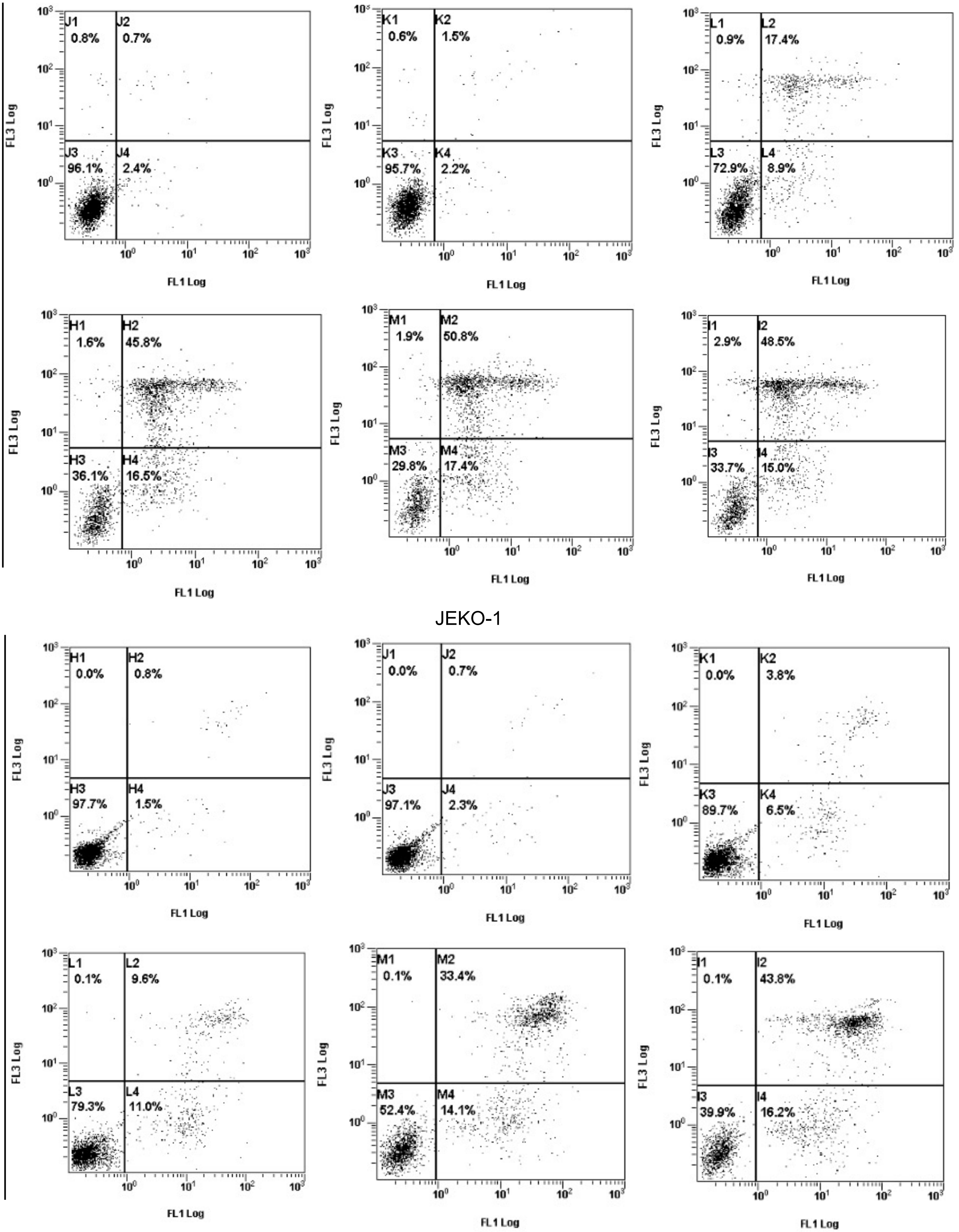

JEKO-1

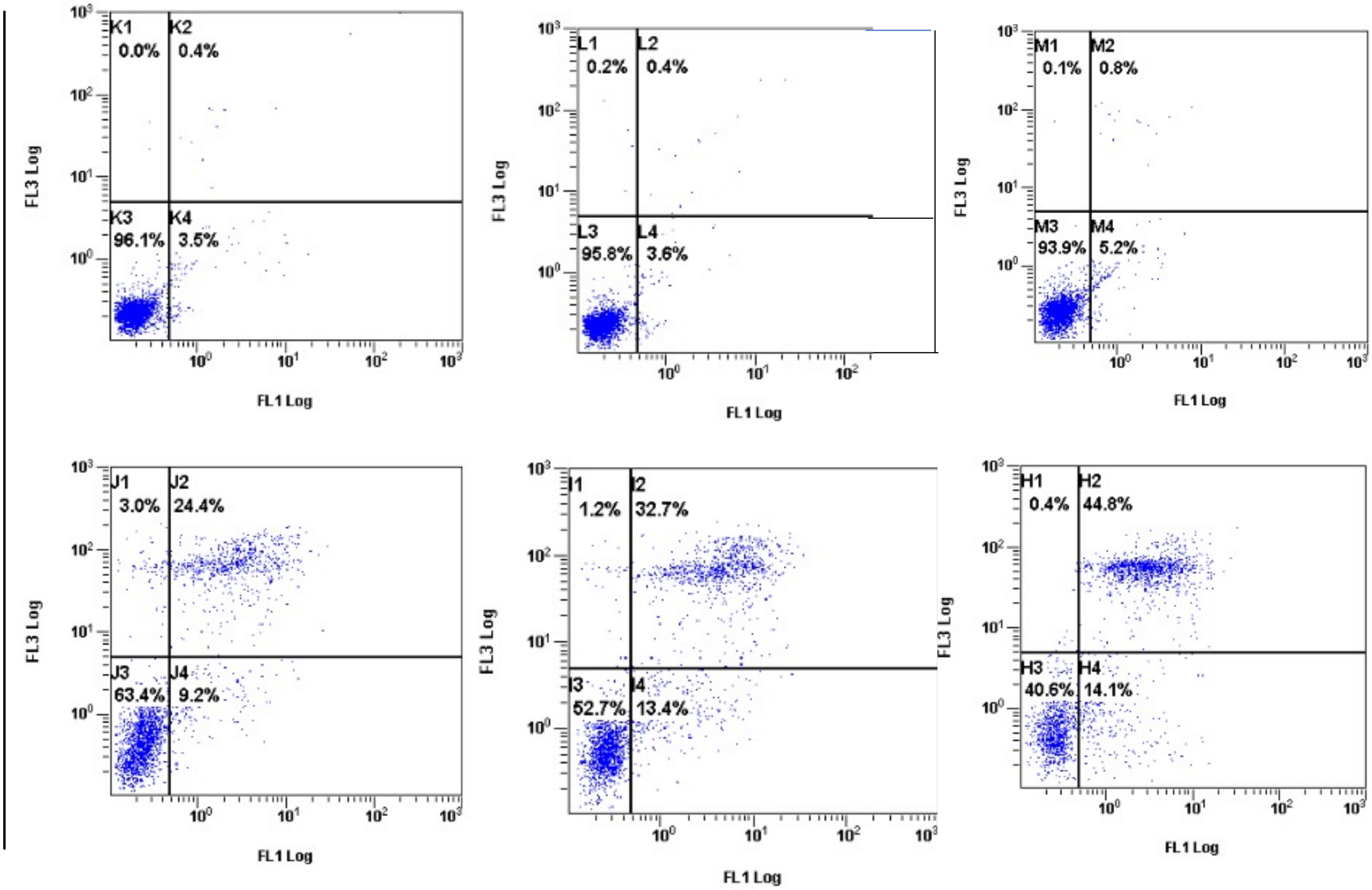
K1 0.0%
K2 0.4%
K3 96.1%
K4 3.5%
L1 0.2%
L2 0.4%
L3 95.8%
L4 3.6%
M1 0.1%
M2 0.8%
M3 93.9%
M4 5.2%
J1 3.0%
J2 24.4%
J3 63.4%
J4 9.2%
I1 1.2%
I2 32.7%
I3 52.7%
I4 13.4%
H1 0.4%
H2 44.8%
H3 40.6%
H4 14.1%
FL3 Log
FL1 Log

Mino

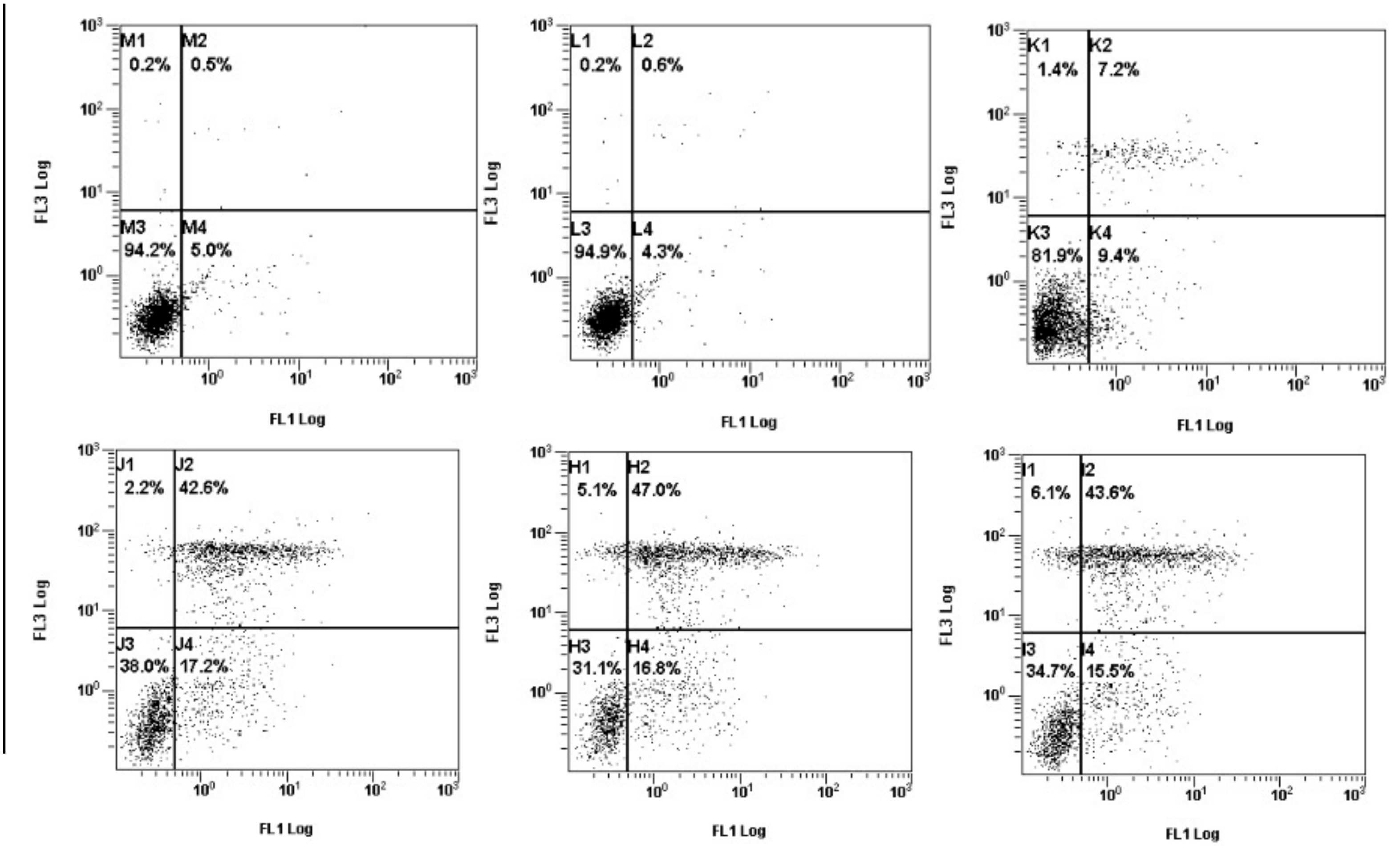

M1 0.2%
M2 0.5%
M3 94.2%
M4 5.0%
L1 0.2%
L2 0.6%
L3 94.9%
L4 4.3%
K1 1.4%
K2 7.2%
K3 81.9%
K4 9.4%
J1 2.2%
J2 42.6%
J3 38.0%
J4 17.2%
H1 5.1%
H2 47.0%
H3 31.1%
H4 16.8%
I1 6.1%
I2 43.6%
I3 34.7%
I4 15.5%
FL3 Log
FL1 Log

Mino

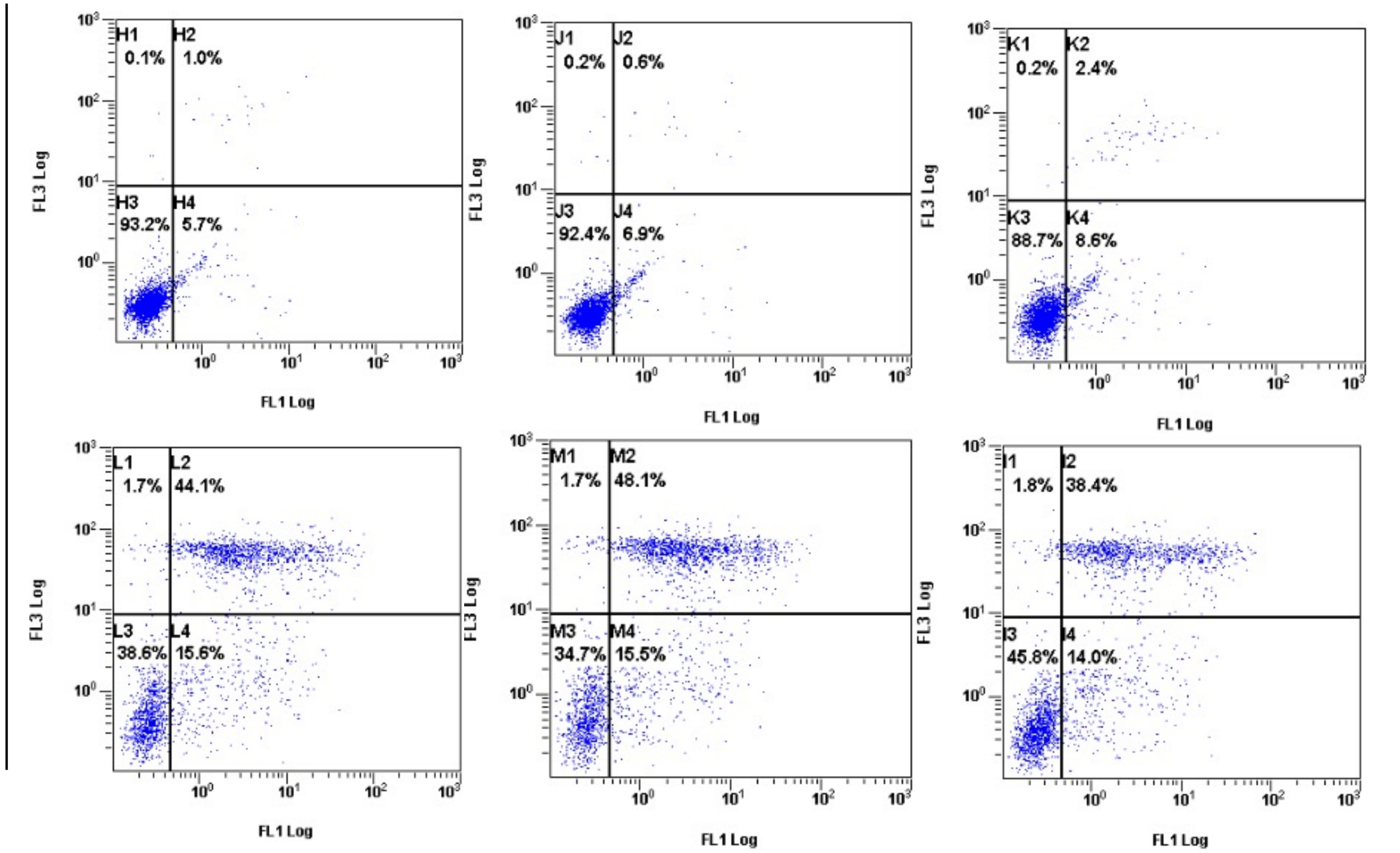

H1 0.1%
H2 1.0%
H3 93.2%
H4 5.7%
J1 0.2%
J2 0.6%
J3 92.4%
J4 6.9%
K1 0.2%
K2 2.4%
K3 88.7%
K4 8.6%
L1 1.7%
L2 44.1%
L3 38.6%
L4 15.6%
M1 1.7%
M2 48.1%
M3 34.7%
M4 15.5%
I1 1.8%
I2 38.4%
I3 45.8%
I4 14.0%
FL3 Log
FL1 Log

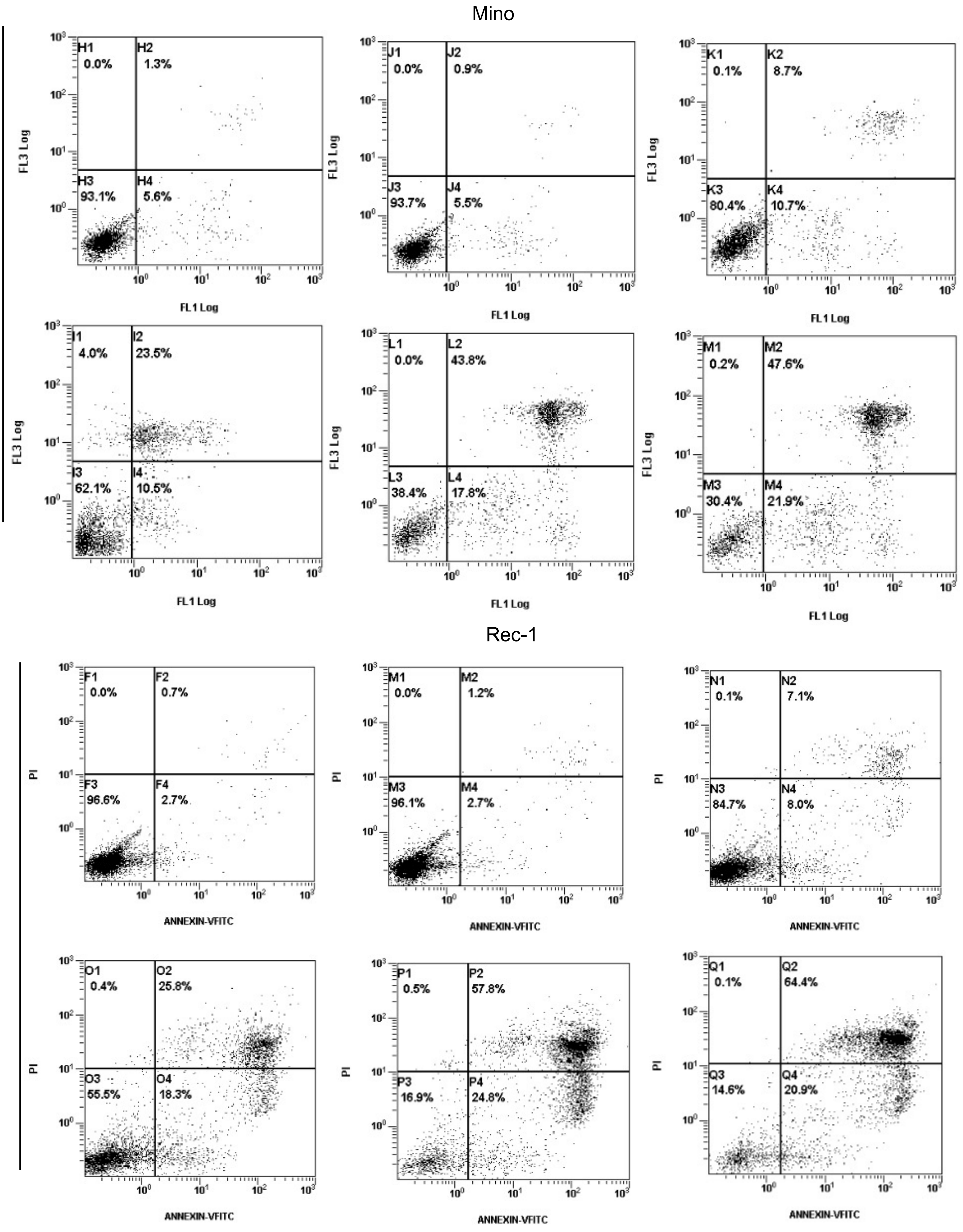
Mino
H1 0.0%
H2 1.3%
H3 93.1%
H4 5.6%
J1 0.0%
J2 0.9%
J3 93.7%
J4 5.5%
K1 0.1%
K2 8.7%
K3 80.4%
K4 10.7%
I1 4.0%
I2 23.5%
I3 62.1%
I4 10.5%
L1 0.0%
L2 43.8%
L3 38.4%
L4 17.8%
M1 0.2%
M2 47.6%
M3 30.4%
M4 21.9%
FL3 Log
FL1 Log
Rec-1
F1 0.0%
F2 0.7%
F3 96.6%
F4 2.7%
M1 0.0%
M2 1.2%
M3 96.1%
M4 2.7%
N1 0.1%
N2 7.1%
N3 84.7%
N4 8.0%
O1 0.4%
O2 25.8%
O3 55.5%
O4 18.3%
P1 0.5%
P2 57.8%
P3 16.9%
P4 24.8%
Q1 0.1%
Q2 64.4%
Q3 14.6%
Q4 20.9%
PI
ANNEXIN-VFITC

Rec-1

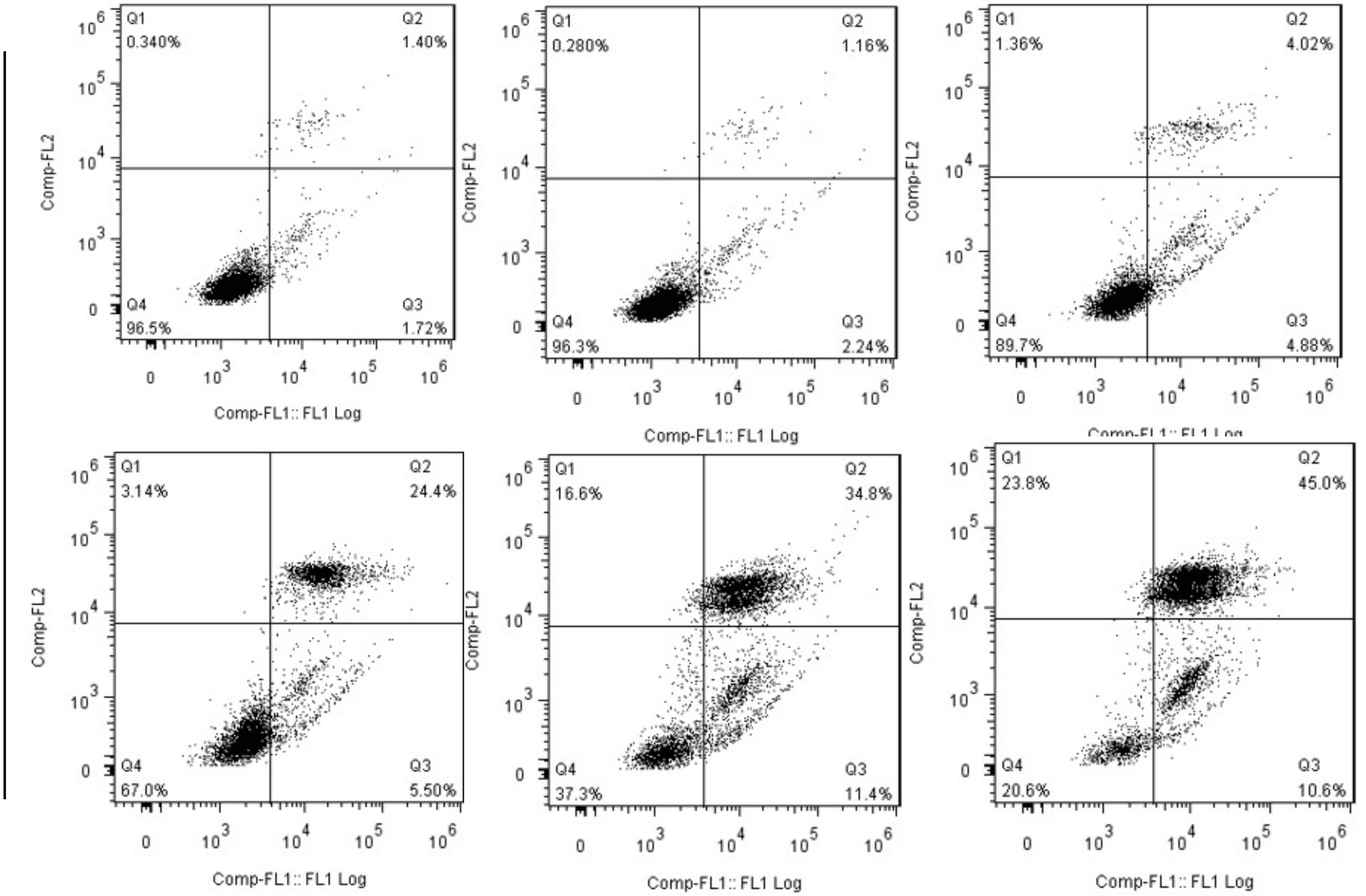
Comp-FL2
Comp-FL1:: FL1 Log
Q1 0.340% Q2 1.40% Q4 96.5% Q3 1.72%
Q1 0.280% Q2 1.16% Q4 96.3% Q3 2.24%
Q1 1.36% Q2 4.02% Q4 89.7% Q3 4.88%
Q1 3.14% Q2 24.4% Q4 67.0% Q3 5.50%
Q1 16.6% Q2 34.8% Q4 37.3% Q3 11.4%
Q1 23.8% Q2 45.0% Q4 20.6% Q3 10.6%

Rec-1

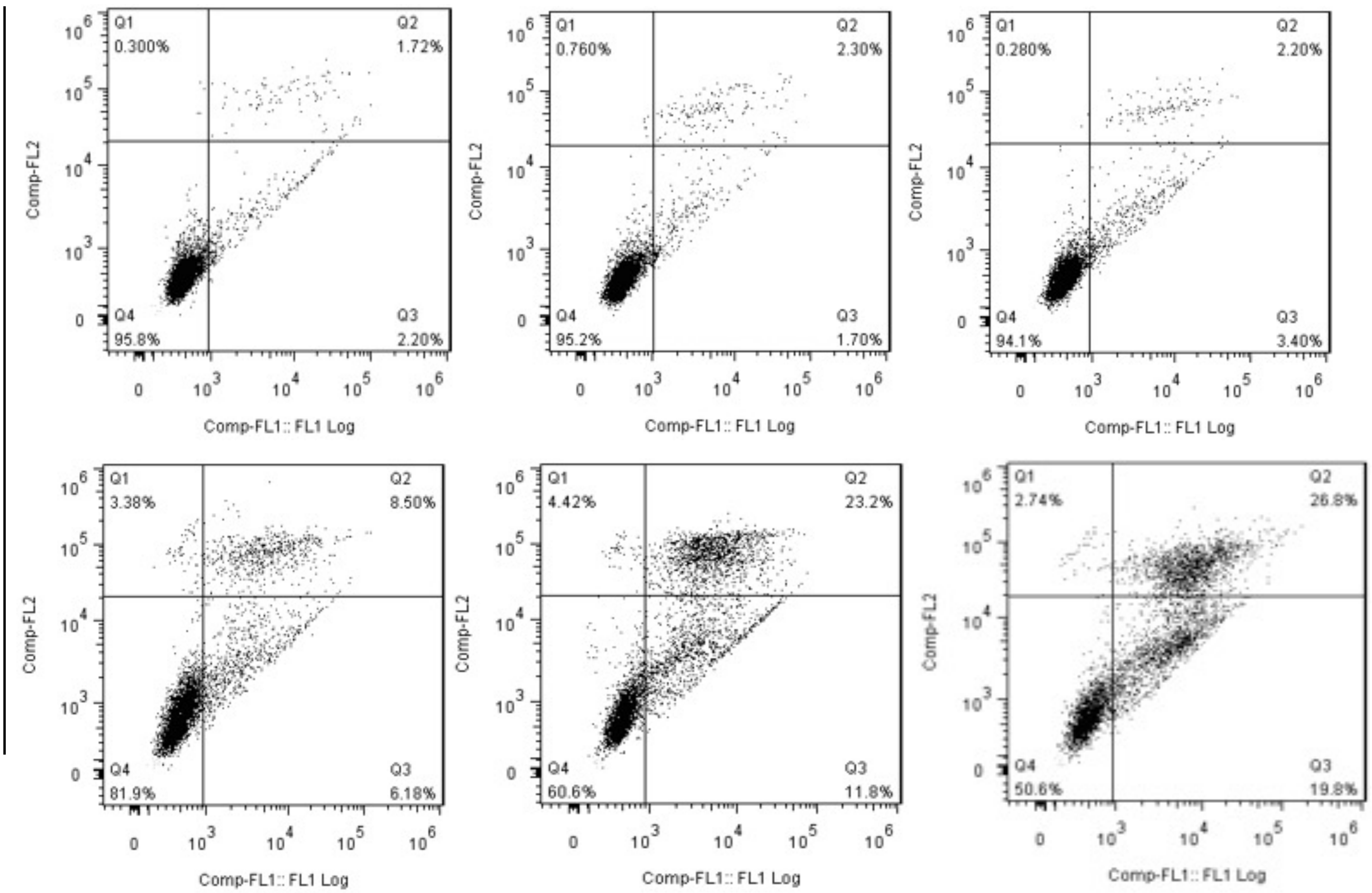
Comp-FL2
Comp-FL1:: FL1 Log
Q1 0.300% Q2 1.72% Q4 95.8% Q3 2.20%
Q1 0.760% Q2 2.30% Q4 95.2% Q3 1.70%
Q1 0.280% Q2 2.20% Q4 94.1% Q3 3.40%
Q1 3.38% Q2 8.50% Q4 81.9% Q3 6.18%
Q1 4.42% Q2 23.2% Q4 60.6% Q3 11.8%
Q1 2.74% Q2 26.8% Q4 50.6% Q3 19.8%

Figure 1C

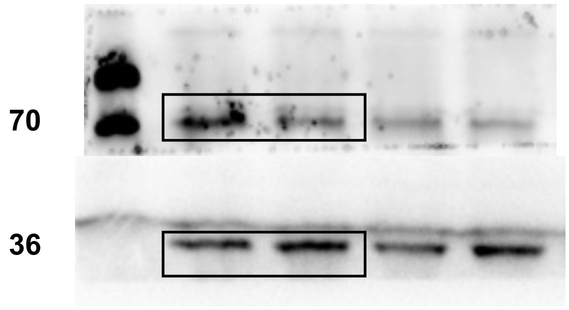

Figure 1D

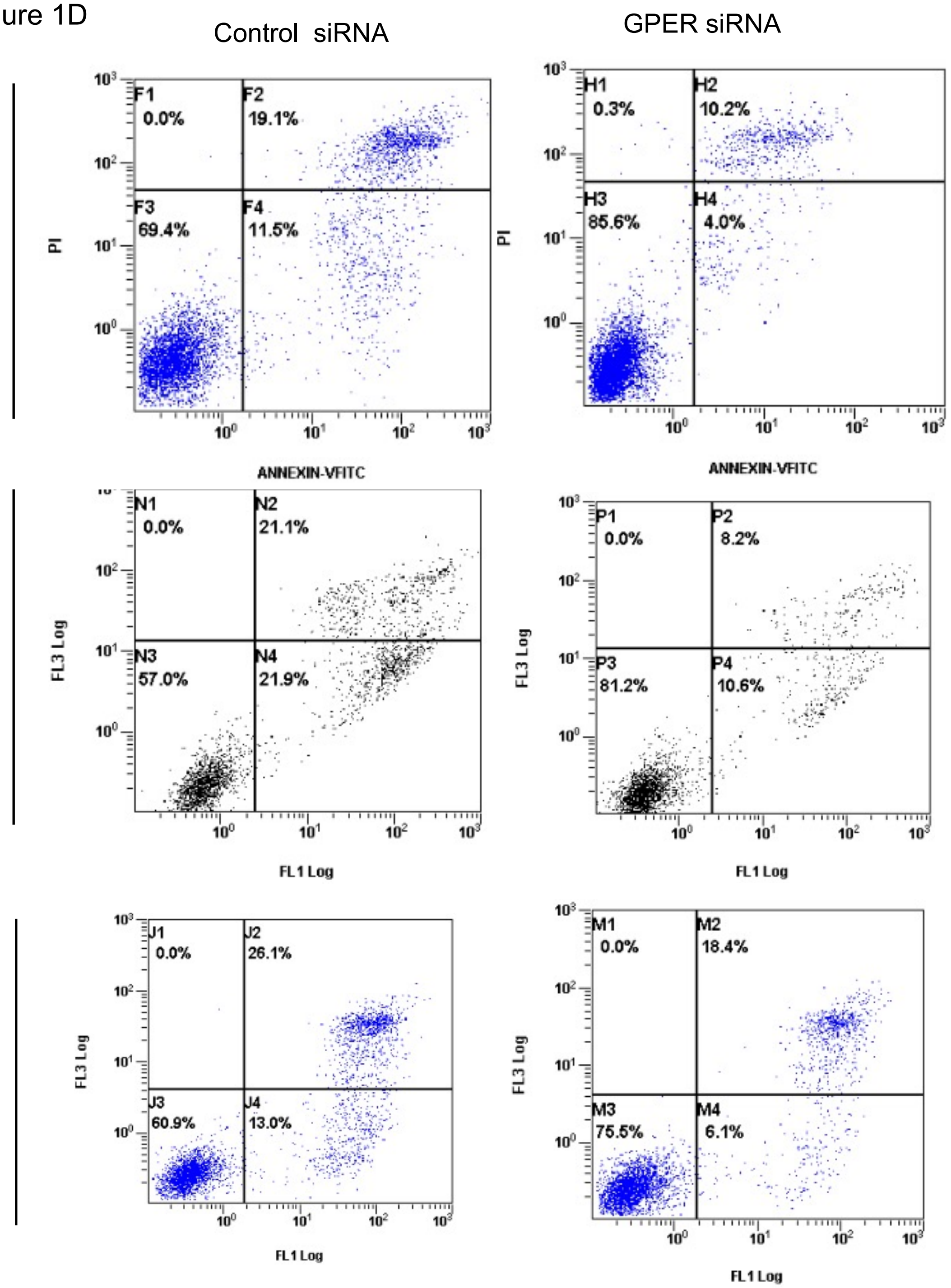

Figure 2B

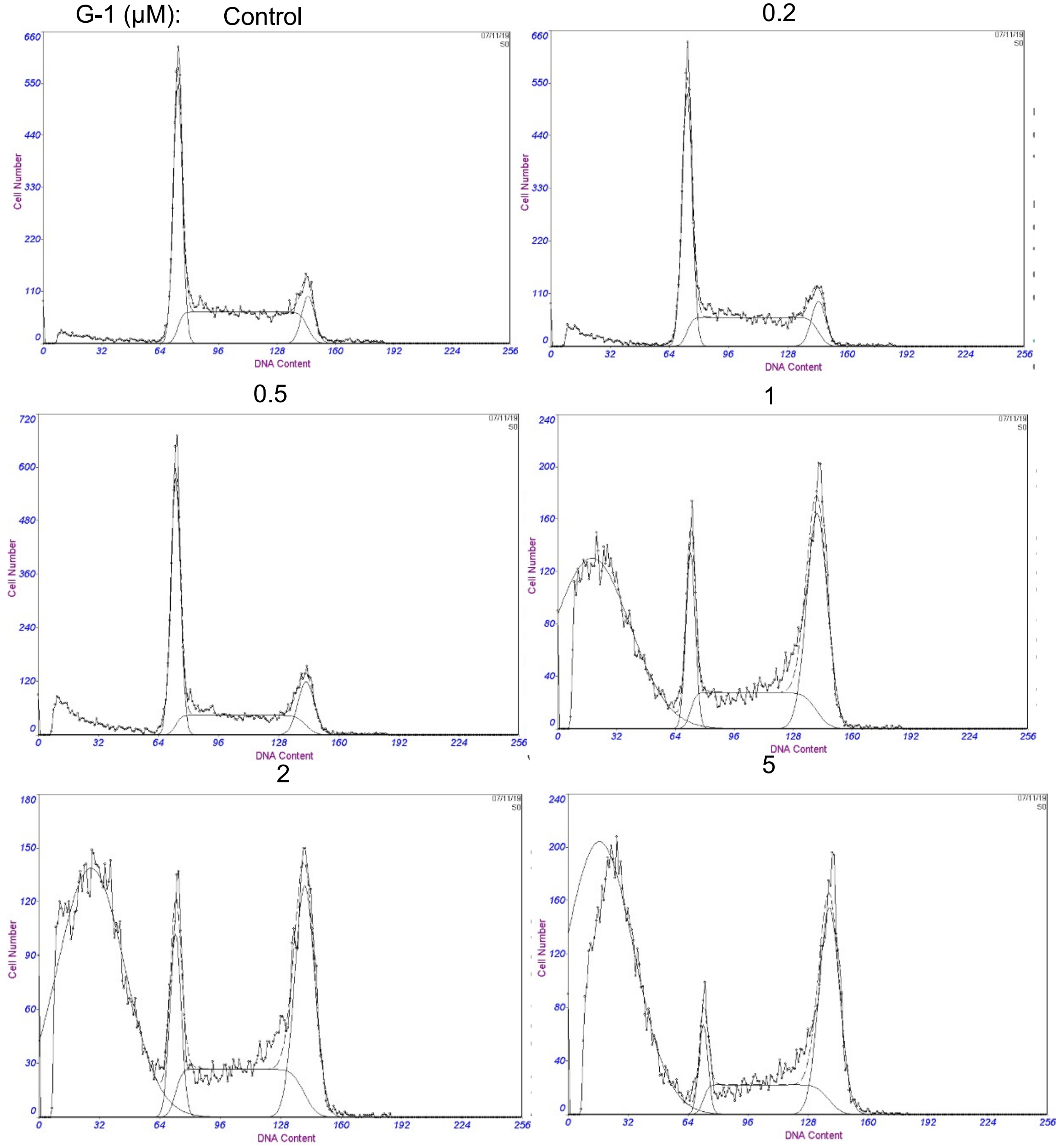

Figure 2D

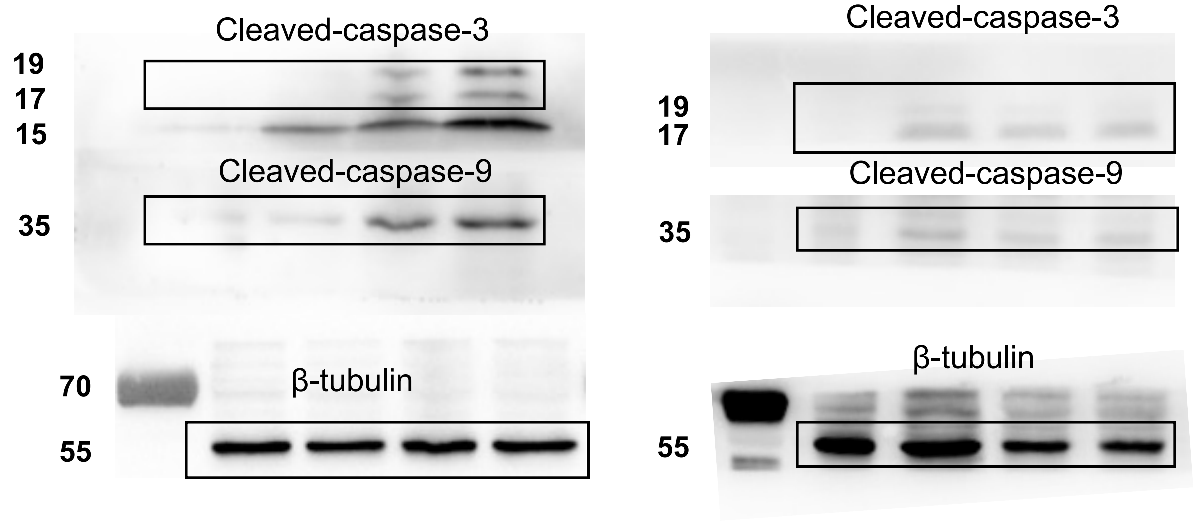

Figure 3B

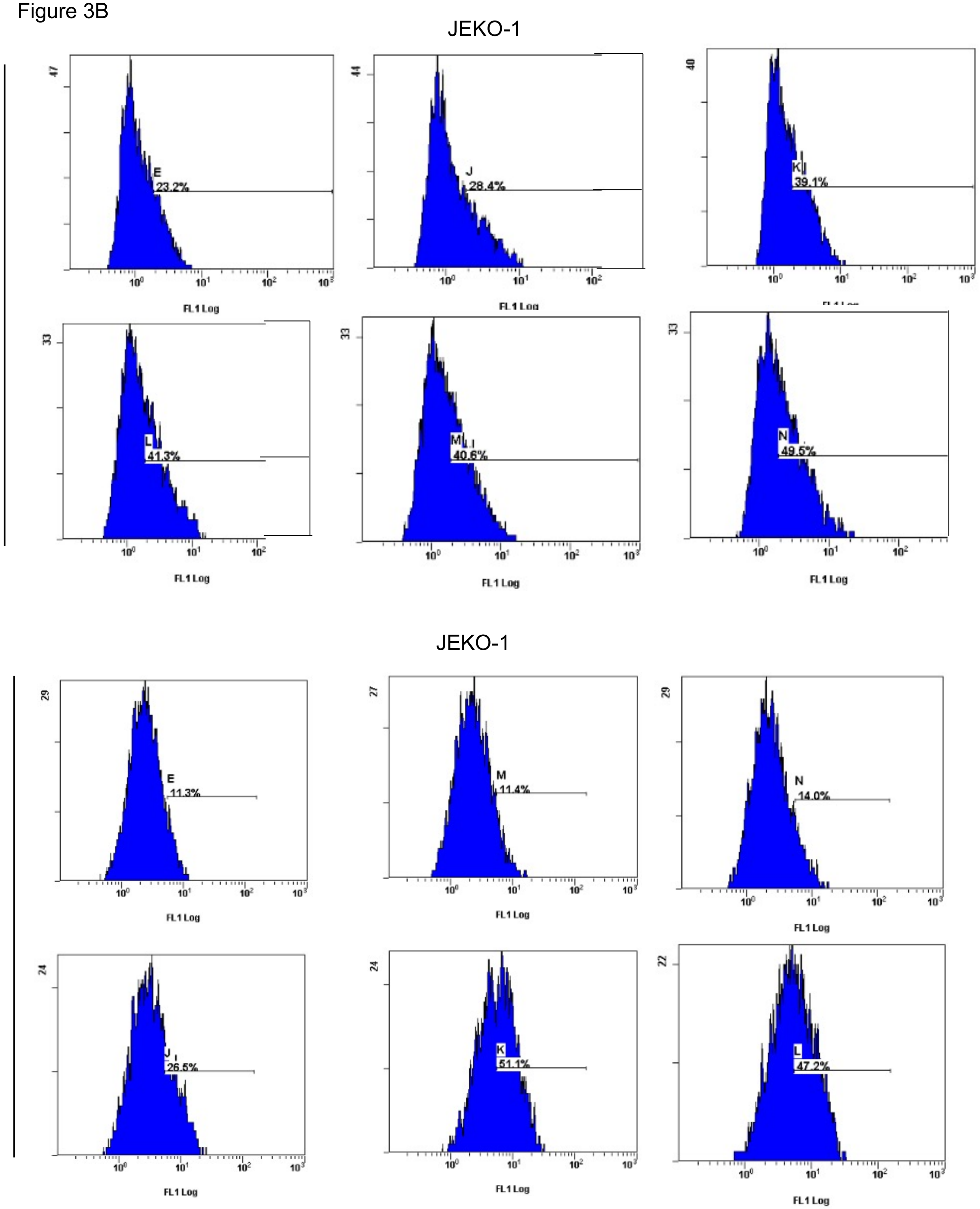

JEKO-1
E
23.2%
J
28.4%
K
39.1%
L
41.3%
M
40.6%
N
49.5%
FL1 Log
JEKO-1
E
11.3%
M
11.4%
N
14.0%
J
26.5%
K
51.1%
L
47.2%
FL1 Log

Figure 3B

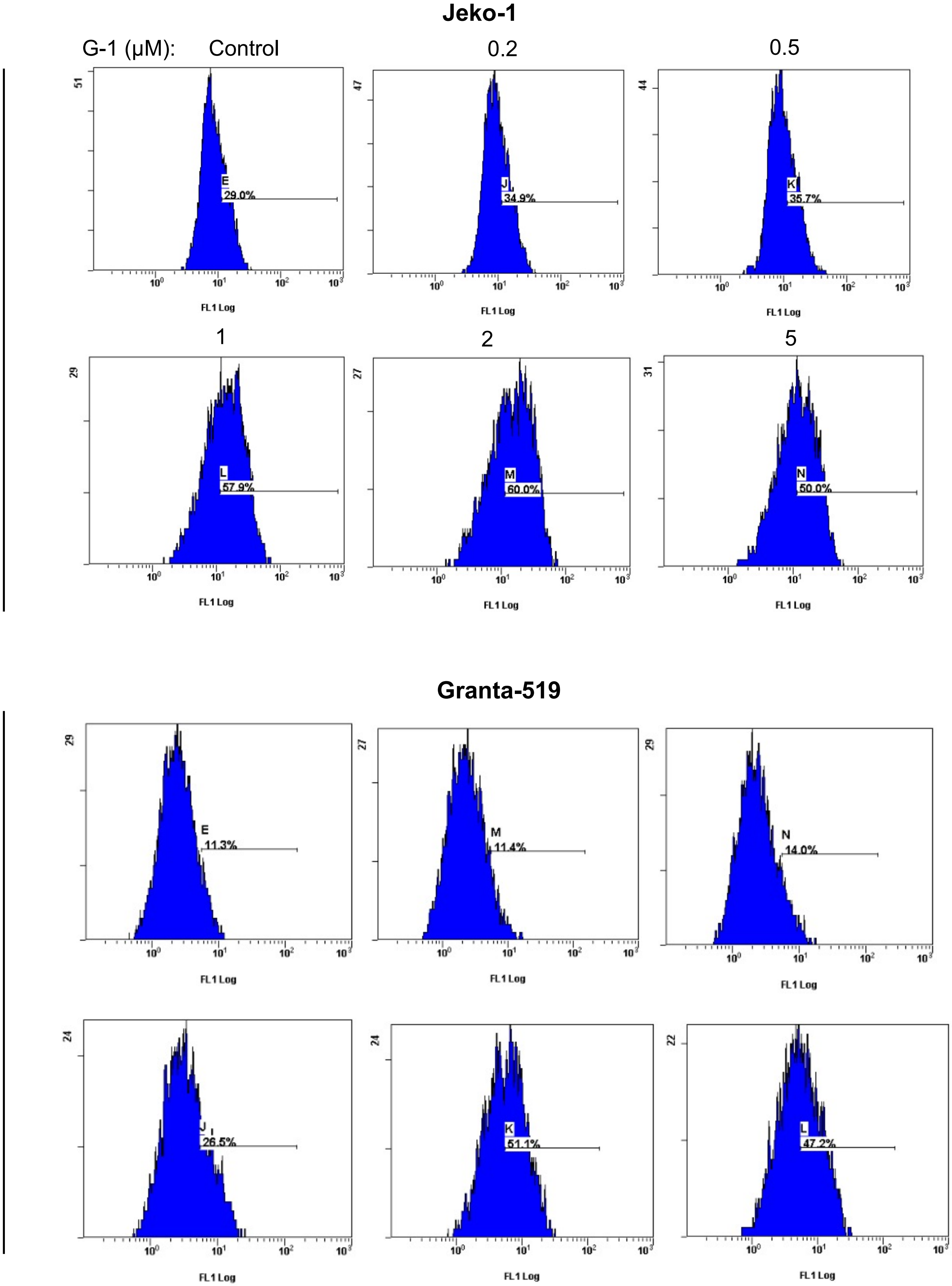
Jeko-1
G-1 (µM): Control
0.2
0.5
1
2
5
E 29.0%
J 34.9%
K 35.7%
L 57.9%
M 60.0%
N 50.0%
FL1 Log
Granta-519
E 11.3%
M 11.4%
N 14.0%
J 26.5%
K 51.1%
L 47.2%

Granta-519

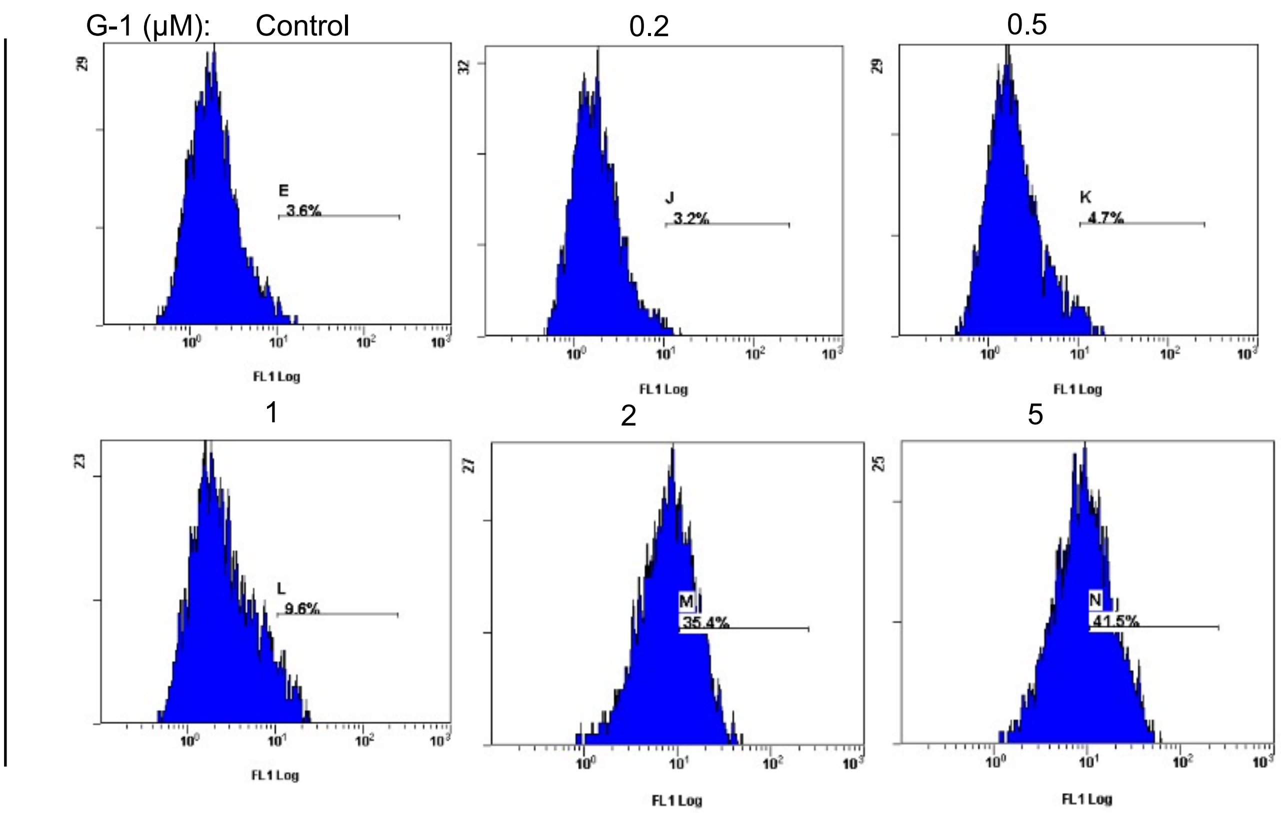

Granta-519

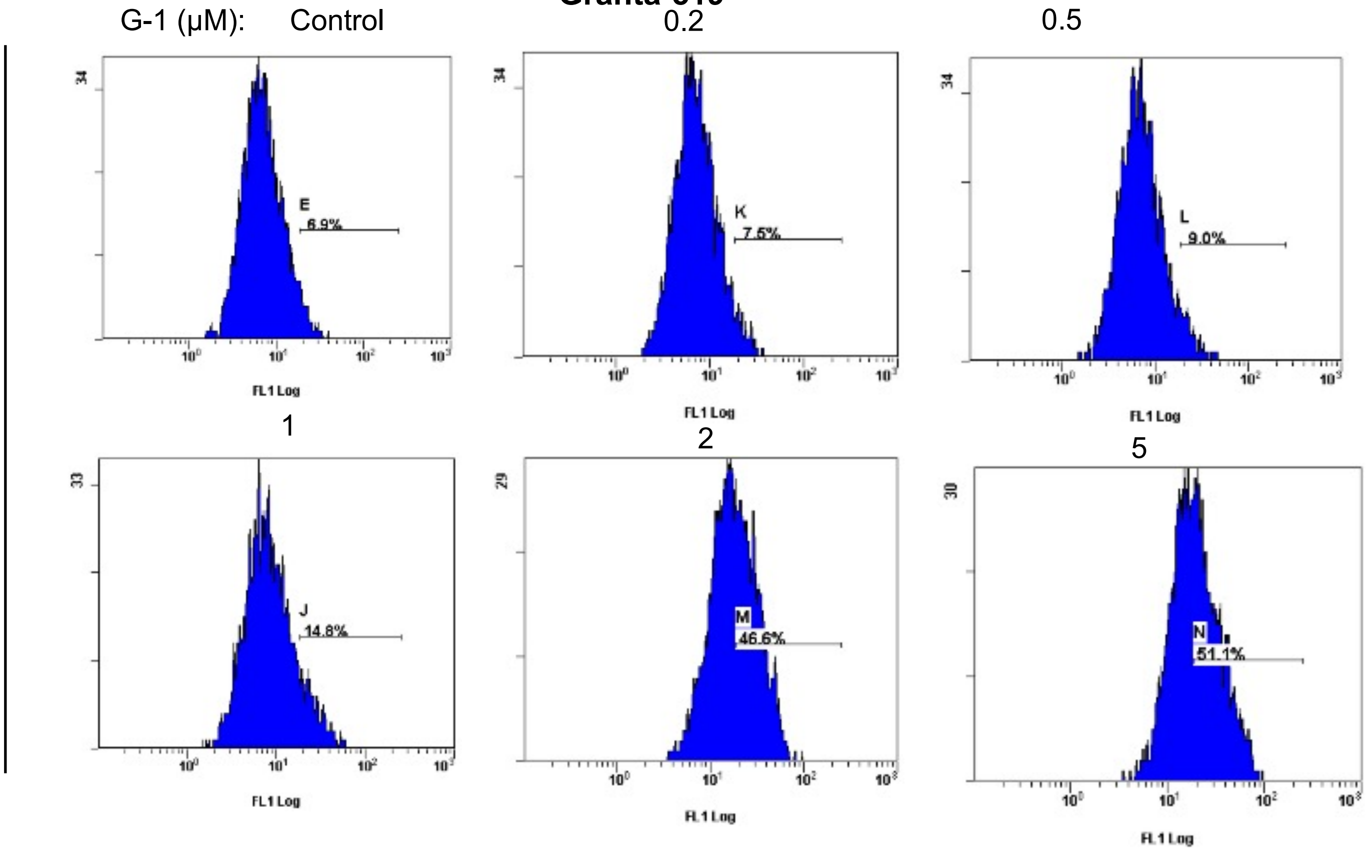

Figure 3C

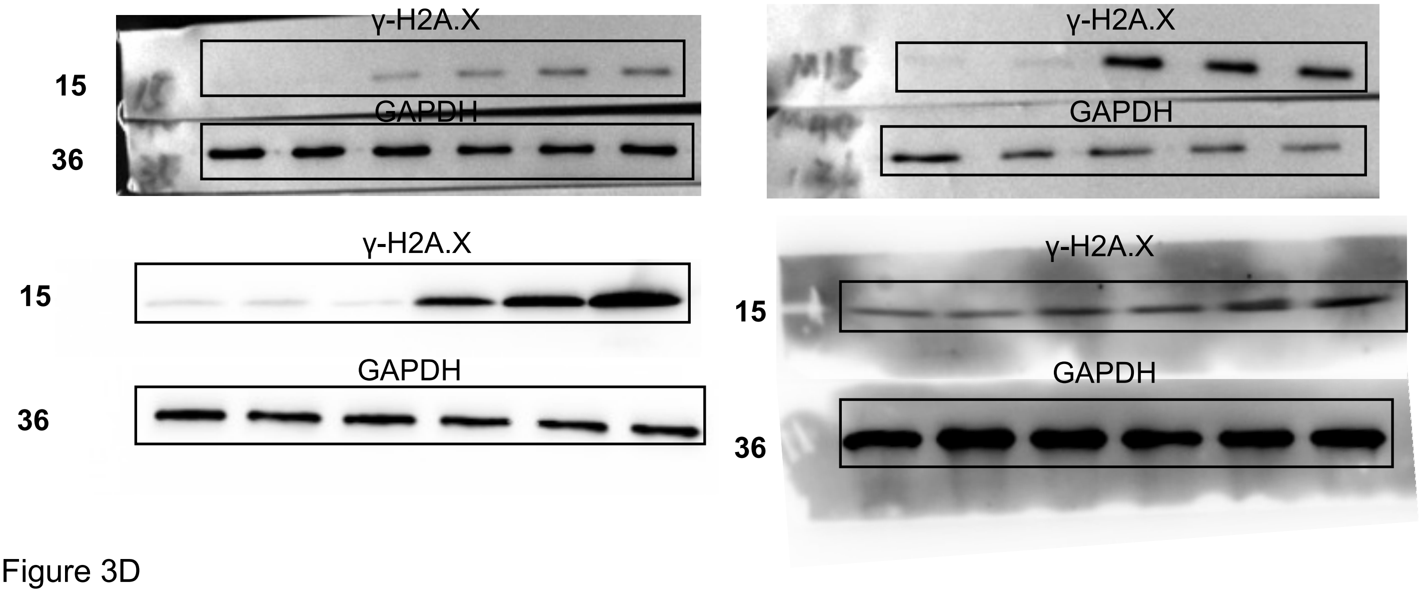

Figure 3D

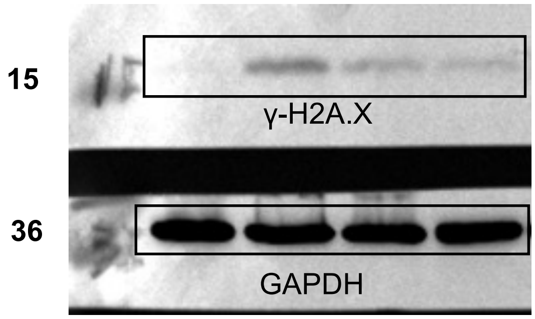

Figure 3E

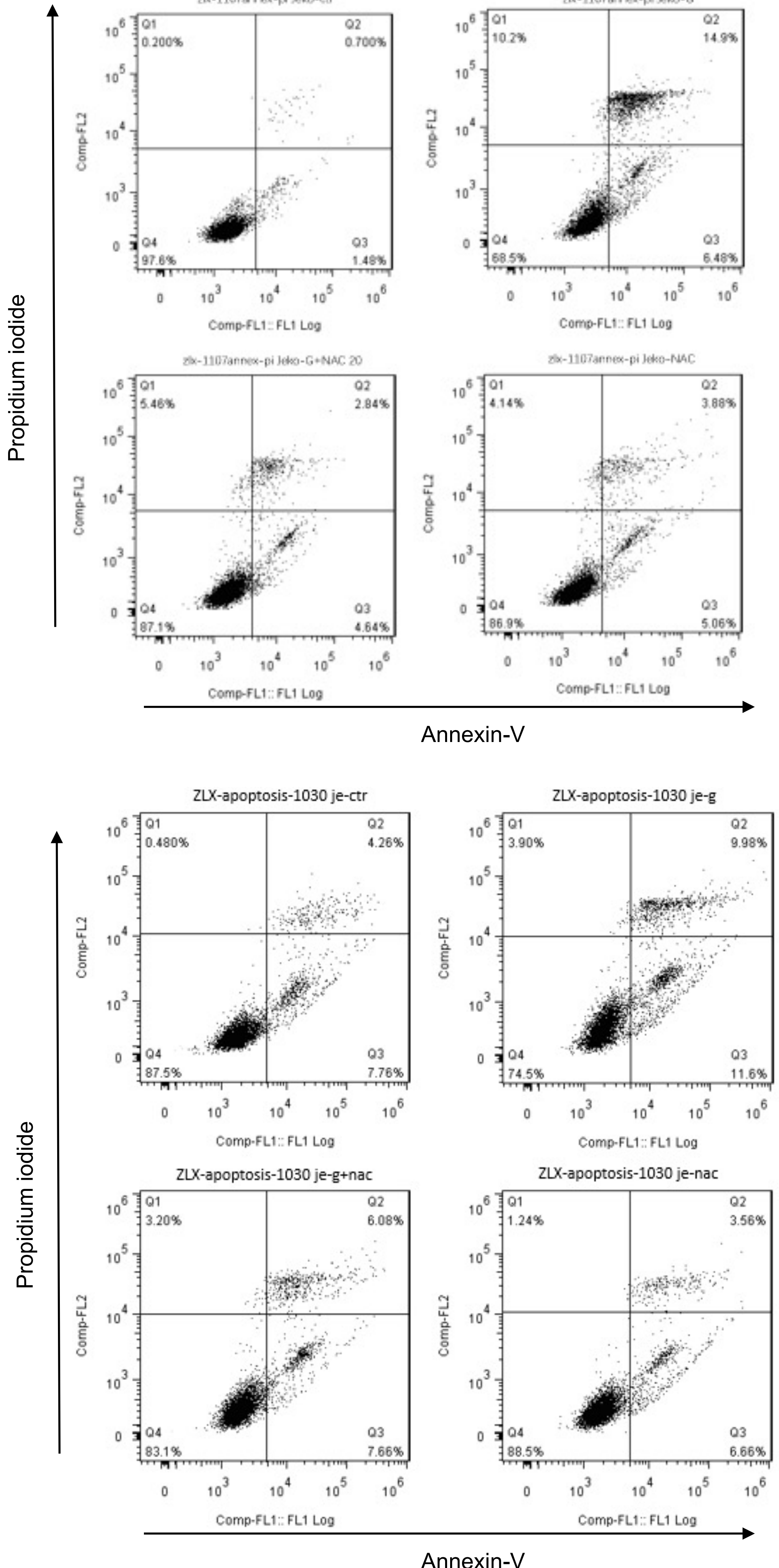

Propidium iodide
Annexin-V
zlx-1107annex-pi Jeko-ctr
Q1 0.200%
Q2 0.700%
Q4 97.6%
Q3 1.48%
zlx-1107annex-pi Jeko-G
Q1 10.2%
Q2 14.9%
Q4 68.5%
Q3 6.48%
zlx-1107annex-pi Jeko-G+NAC 20
Q1 5.46%
Q2 2.84%
Q4 87.1%
Q3 4.64%
zlx-1107annex-pi Jeko-NAC
Q1 4.14%
Q2 3.88%
Q4 86.9%
Q3 5.06%
ZLX-apoptosis-1030 je-ctr
Q1 0.480%
Q2 4.26%
Q4 87.5%
Q3 7.76%
ZLX-apoptosis-1030 je-g
Q1 3.90%
Q2 9.98%
Q4 74.5%
Q3 11.6%
ZLX-apoptosis-1030 je-g+nac
Q1 3.20%
Q2 6.08%
Q4 83.1%
Q3 7.66%
ZLX-apoptosis-1030 je-nac
Q1 1.24%
Q2 3.56%
Q4 88.5%
Q3 6.66%
Comp-FL2
Comp-FL1:: FL1 Log

Figure 3E

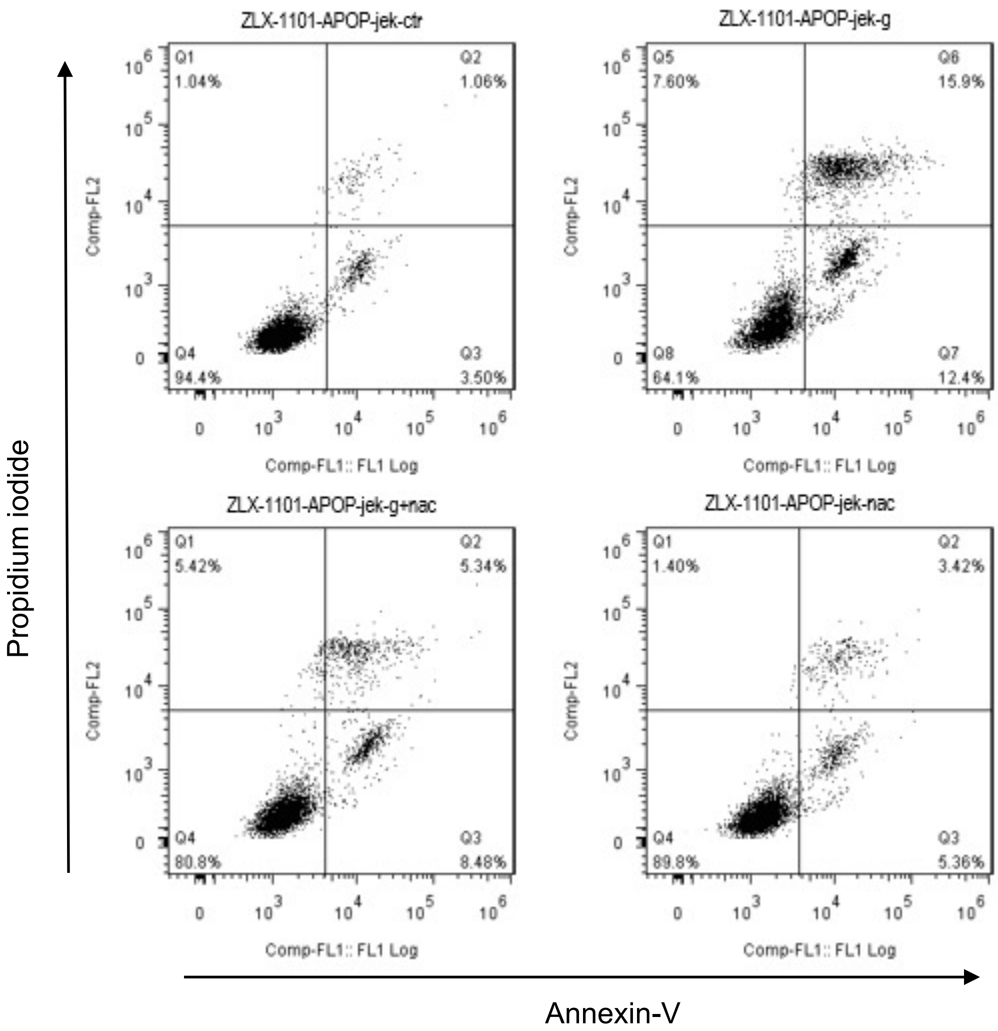
ZLX-1101-APOP-jek-ctr
Q1 1.04%
Q2 1.06%
Q4 94.4%
Q3 3.50%
ZLX-1101-APOP-jek-g
Q5 7.60%
Q6 15.9%
Q8 64.1%
Q7 12.4%
ZLX-1101-APOP-jek-g+nac
Q1 5.42%
Q2 5.34%
Q4 80.8%
Q3 8.48%
ZLX-1101-APOP-jek-nac
Q1 1.40%
Q2 3.42%
Q4 89.8%
Q3 5.36%
Comp-FL2
Comp-FL1:: FL1 Log
Propidium iodide
Annexin-V

Figure 4A

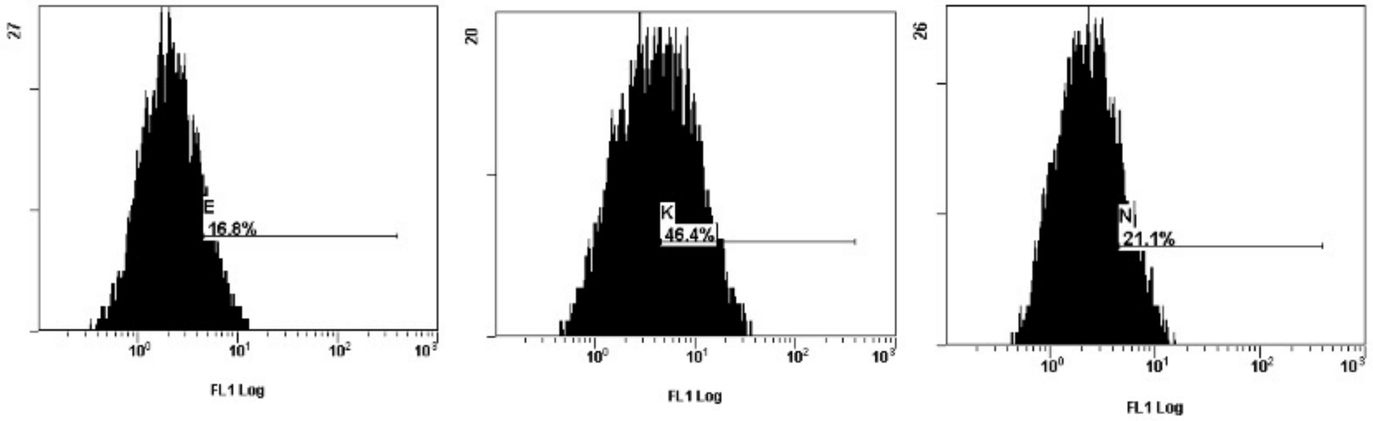

Figure 4B

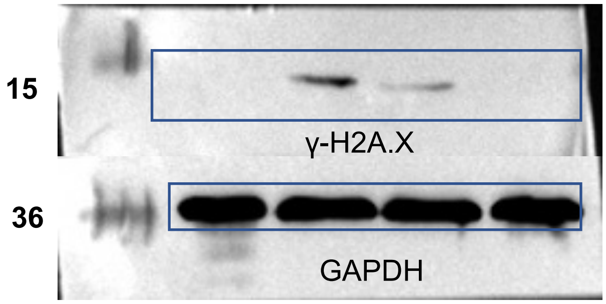

Figure 4C

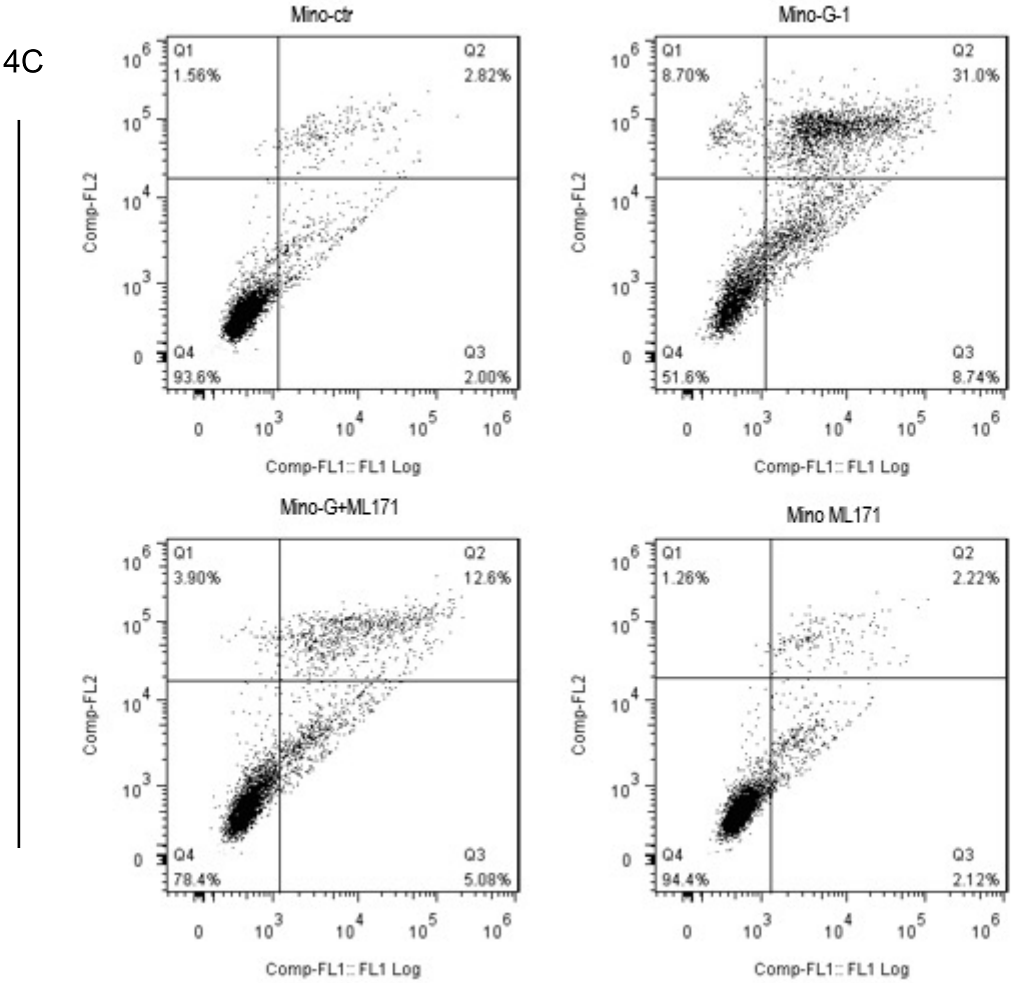

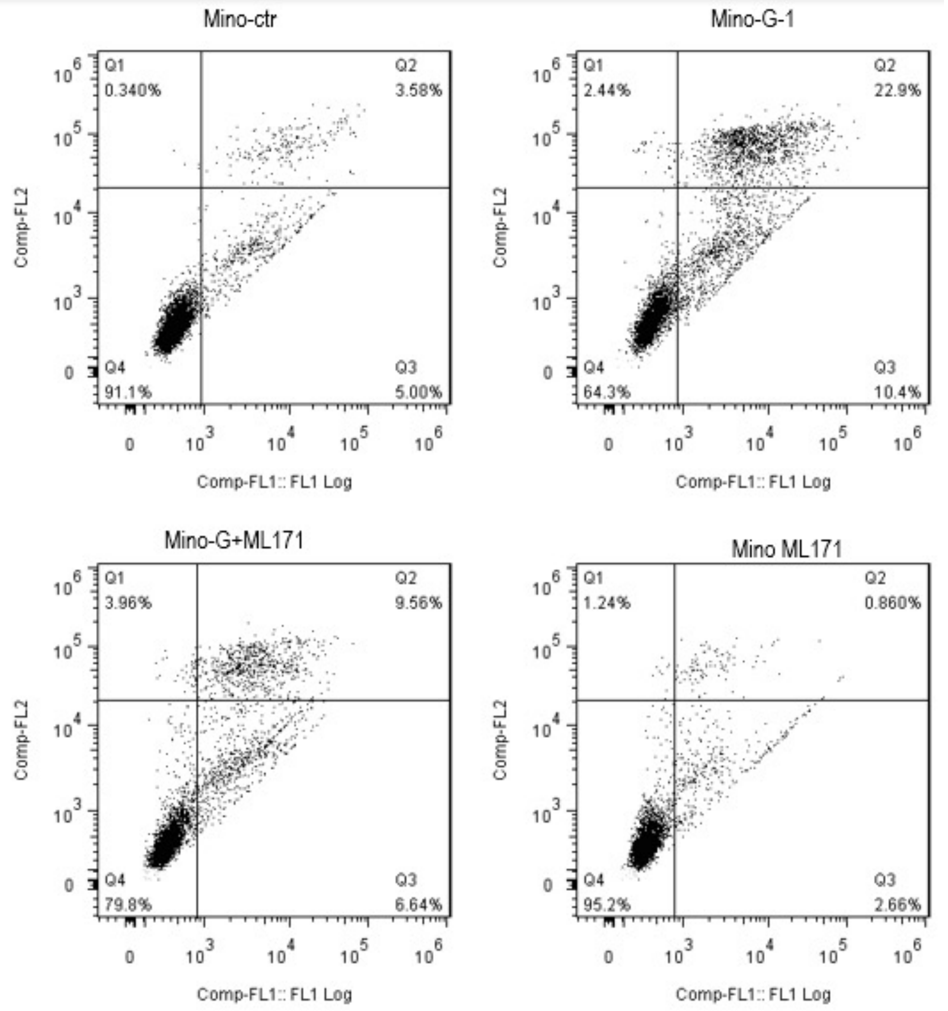
Mino-ctr
Q1 0.340%
Q2 3.58%
Q4 91.1%
Q3 5.00%
Mino-G-1
Q1 2.44%
Q2 22.9%
Q4 64.3%
Q3 10.4%
Mino-G+ML171
Q1 3.96%
Q2 9.56%
Q4 79.8%
Q3 6.64%
Mino ML171
Q1 1.24%
Q2 0.860%
Q4 95.2%
Q3 2.66%
Comp-FL2
Comp-FL1:: FL1 Log

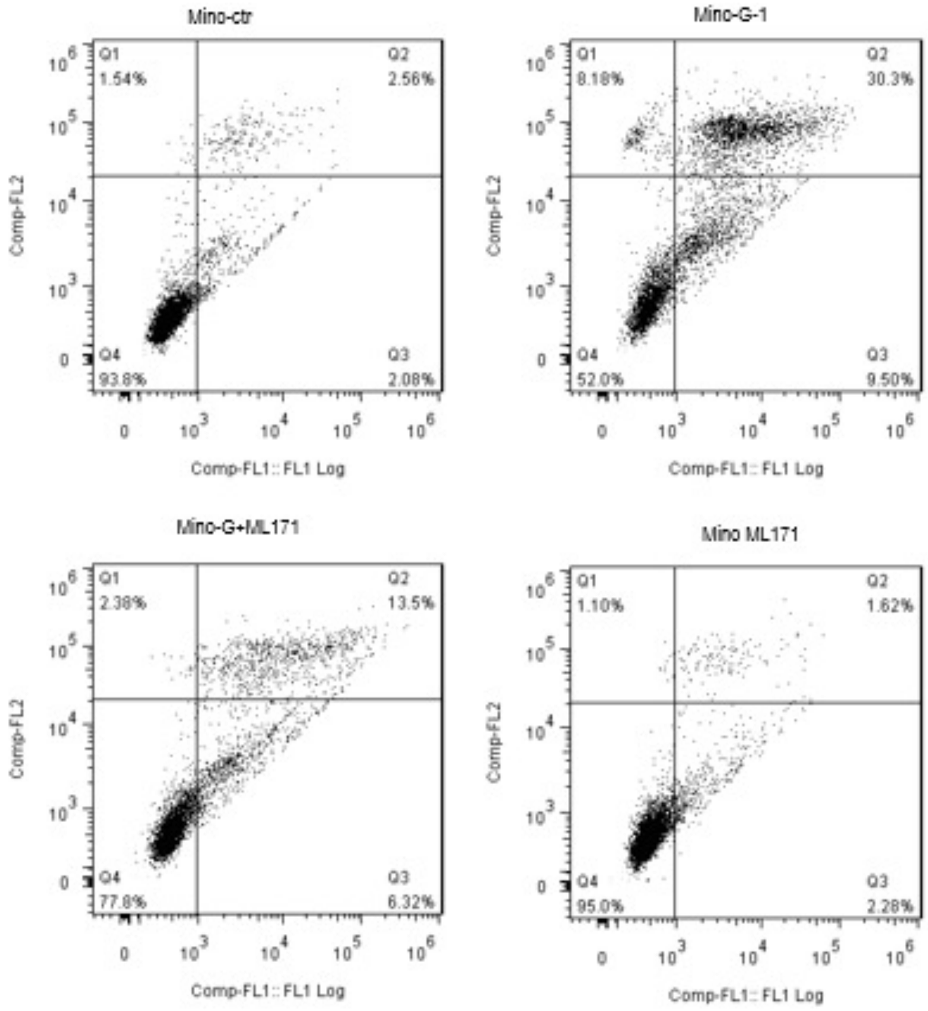
Mino-ctr
Q1 1.54%
Q2 2.56%
Q4 93.8%
Q3 2.08%
Mino-G-1
Q1 8.18%
Q2 30.3%
Q4 52.0%
Q3 9.50%
Mino-G+ML171
Q1 2.38%
Q2 13.5%
Q4 77.8%
Q3 6.32%
Mino ML171
Q1 1.10%
Q2 1.62%
Q4 95.0%
Q3 2.28%
Comp-FL2
Comp-FL1:: FL1 Log

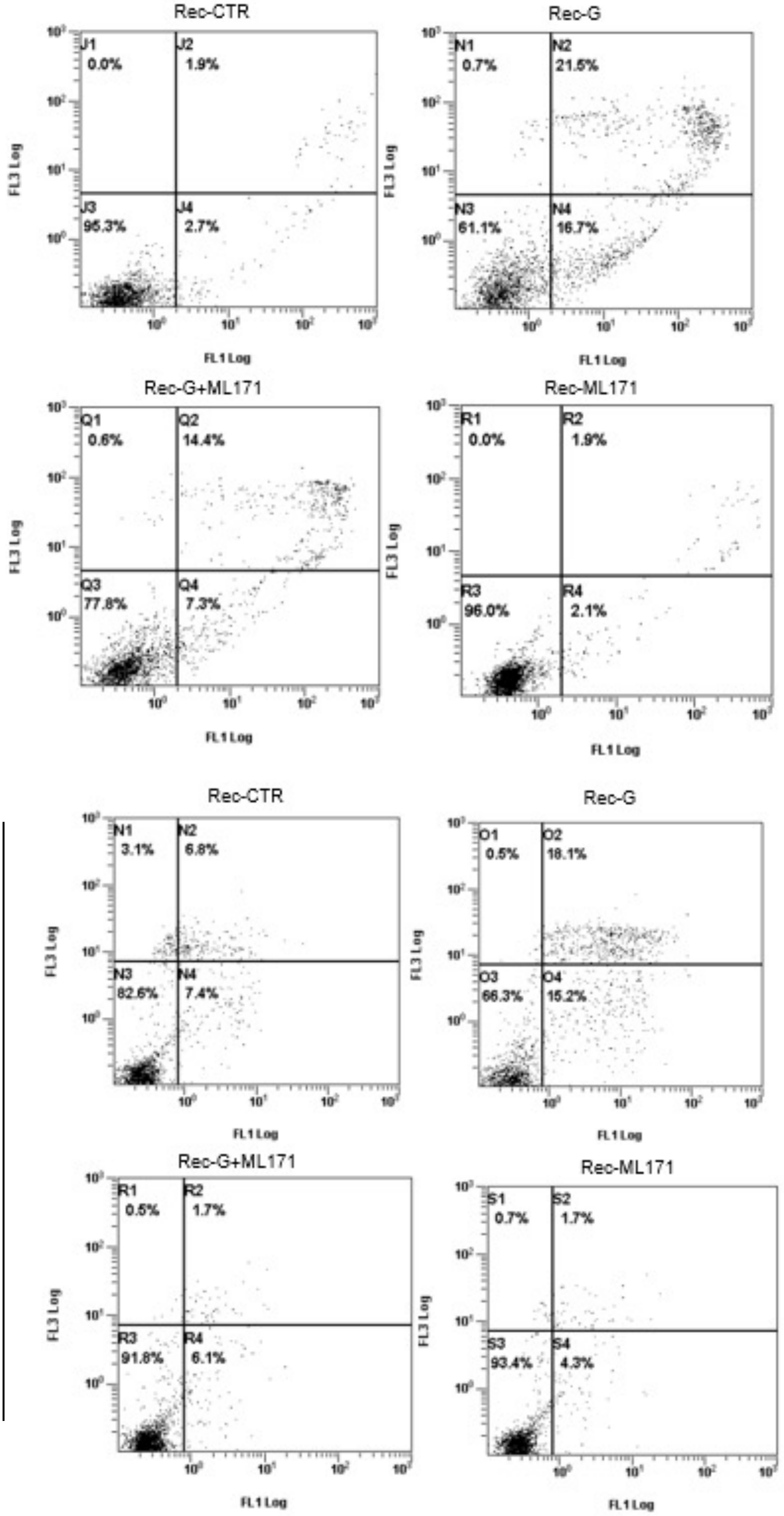
Rec-CTR
J1 0.0%
J2 1.9%
J3 95.3%
J4 2.7%
Rec-G
N1 0.7%
N2 21.5%
N3 61.1%
N4 16.7%
Rec-G+ML171
Q1 0.6%
Q2 14.4%
Q3 77.8%
Q4 7.3%
Rec-ML171
R1 0.0%
R2 1.9%
R3 96.0%
R4 2.1%
Rec-CTR
N1 3.1%
N2 6.8%
N3 82.6%
N4 7.4%
Rec-G
O1 0.5%
O2 18.1%
O3 66.3%
O4 15.2%
Rec-G+ML171
R1 0.5%
R2 1.7%
R3 91.8%
R4 6.1%
Rec-ML171
S1 0.7%
S2 1.7%
S3 93.4%
S4 4.3%
FL3 Log
FL1 Log

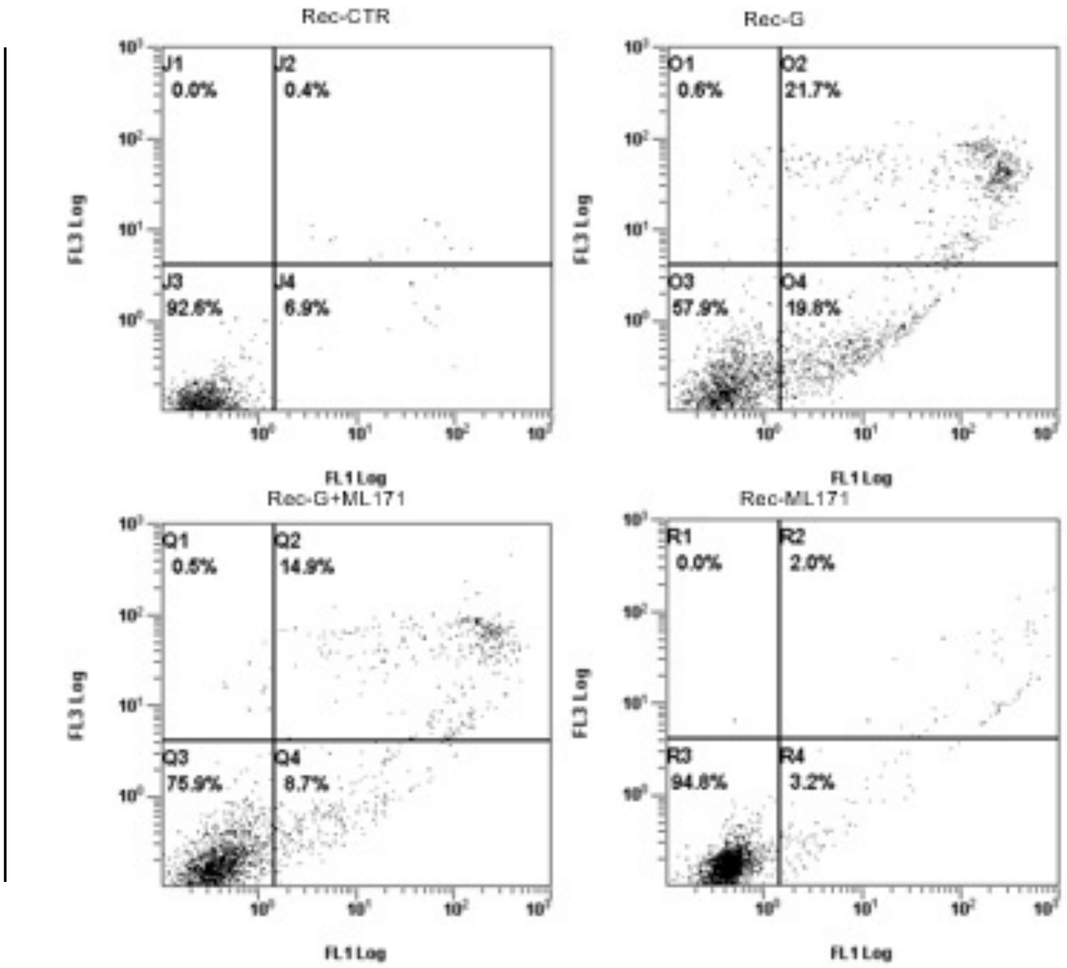
Rec-CTR
J1 0.0%
J2 0.4%
J3 92.6%
J4 6.9%
Rec-G
O1 0.6%
O2 21.7%
O3 57.9%
O4 19.8%
Rec-G+ML171
Q1 0.5%
Q2 14.9%
Q3 75.9%
Q4 8.7%
Rec-ML171
R1 0.0%
R2 2.0%
R3 94.8%
R4 3.2%
FL3 Log
FL1 Log

Figure 4D

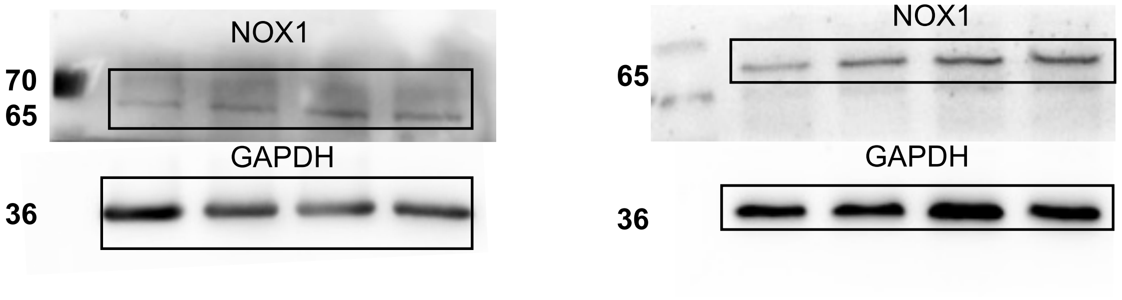
NOX1
70
65
GAPDH
36
NOX1
65
GAPDH
36

Figure 5A

JEKO-1 OD value	blank	DMSO	G-1 0.04uM	G-1 0.1uM	G-1 0.2uM	G-1 0.5uM	G-1 1uM	G-1 2uM	G-1 4uM	G-1 8uM
72h	0.122	1.085	0.998	0.958	0.981	0.789	0.387	0.19	0.158	0.162
	0.121	1.104	0.989	0.991	0.965	0.822	0.373	0.193	0.173	0.177
	0.124	1.29	0.98	0.966	0.979	0.8	0.375	0.213	0.186	0.192
Mino OD value	0.225	1.516	1.444	1.436	1.468	0.985	0.295	0.235	0.251	0.226
72h	0.139	1.384	1.397	1.487	1.471	0.935	0.235	0.172	0.172	0.175
	0.194	1.516	1.442	1.438	1.419	0.976	0.284	0.214	0.226	0.24
Rec-1 OD value	0.146	0.876	0.851	0.821	0.832	0.774	0.524	0.424	0.355	0.381
72h	0.143	0.899	0.842	0.8	0.855	0.75	0.538	0.108	0.37	0.359
	0.17	0.903	0.894	0.869	0.865	0.777	0.543	0.43	0.368	0.375
Granta-519 OD value	0.134	1.596	1.579	1.553	1.603	1.441	0.927	0.481	0.358	0.343
72h	0.125	1.583	1.563	1.434	1.564	1.444	0.962	0.477	0.359	0.349
	0.14	1.578	1.575	1.491	1.502	1.411	0.985	0.484	0.363	0.348

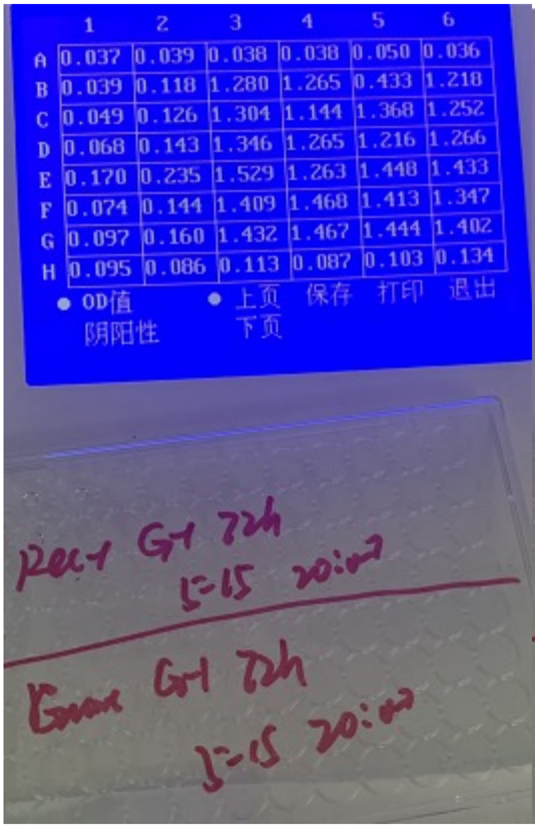

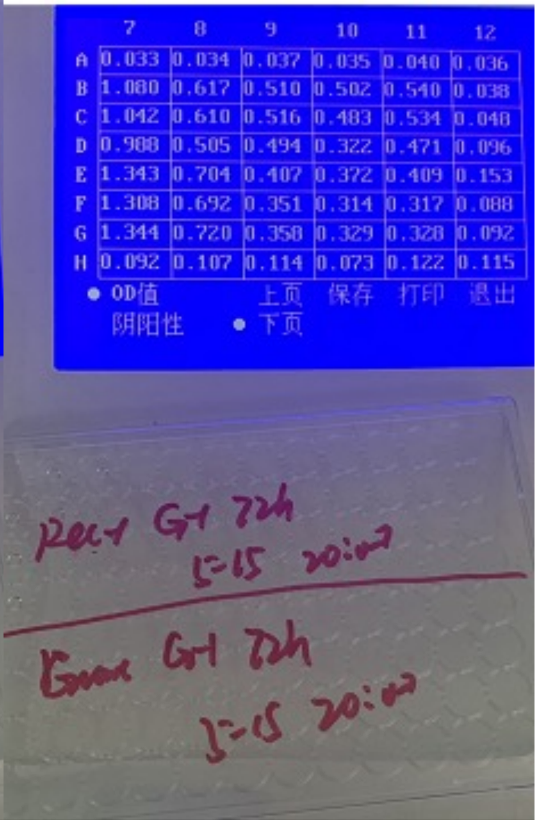

Figure 5B

JEKO-1	Blank	DMSO	IB	G-1	IB/G-1
	0.155	1.102	0.575	0.681	0.352
	0.063	1.132	0.6	0.608	0.338
	0.115	1.137	0.553	0.688	0.457
Mino	blank	DMSO	IB	G-1	IB/G-1
	0.108	0.735	0.382	0.274	0.15
	0.117	0.782	0.427	0.225	0.163
	0.11	0.872	0.386	0.242	0.177

Figure 5C

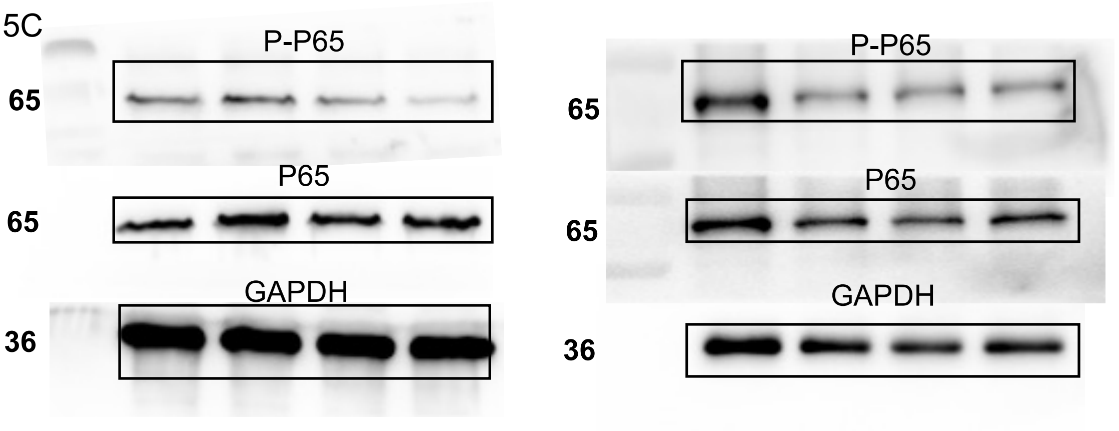

Figure 6A

time	mice	short(mm)	length(mm)
Day 1	1	2	3
	2	3	3
	3	3	3
	4	3	3
	5	3	3
	6	2	3
	7	3	3
	8	3	3
	9	3	3
	10	3	3
Day 3	1	3	4
	2	4	5
	3	4	4
	4	3	4
	5	3	4
	6	3	3
	7	3	3
	8	3.5	4
	9	3	4
	10	4	4
Day 5	1	3	5
	2	6	7
	3	5	7
	4	3	5
	5	4	5
	6	3	4
	7	4	5
	8	5	5
	9	3	4
	10	4	4
Day 7	1	8	8
	2	8	10
	3	9	10
	4	8	10
	5	7	10
	6	4	5
	7	6	7
	8	6	7
	9	5	7
	10	6	6
Day 9	1	9	12
	2	9	14
	3	11	15
	4	11	14
	5	10	12
	6	6	6
	7	9	9
	8	9	9
	9	8	8
	10	9	9
Day 11	1	9	13.5
	2	11	16
	3	11	16
	4	11	17
	5	13	13.5
	6	6	7
	7	10	12
	8	9	10
	9	7	8
	10	10	10
Day 13	1	12	15
	2	12	17.5
	3	12	17
	4	13	19
	5	14	17
	6	8	8.5
	7	11	12
	8	10	12
	9	8	8
	10	10	12.5

Figure 6B

mice	weight(g)
1	0.71
2	0.9
3	0.853
4	1.02
5	0.95
6	0.22
7	0.57
8	0.38
9	0.3
10	0.5

Sup Figure 1A

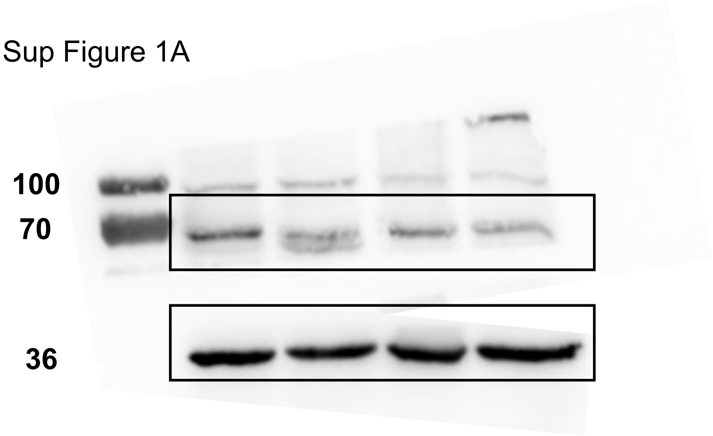

Sup Figure 1B

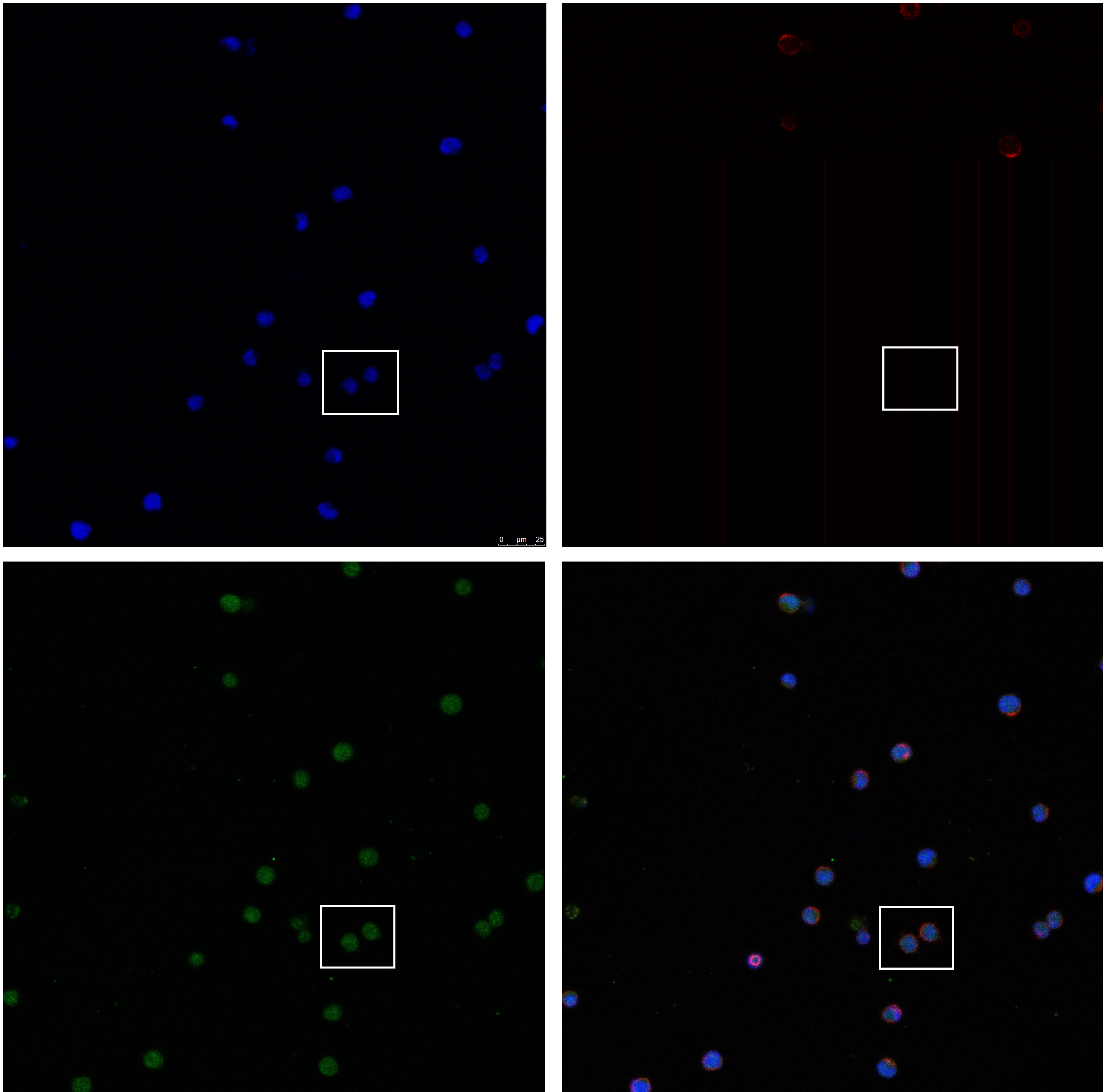

Sup Figure 1C

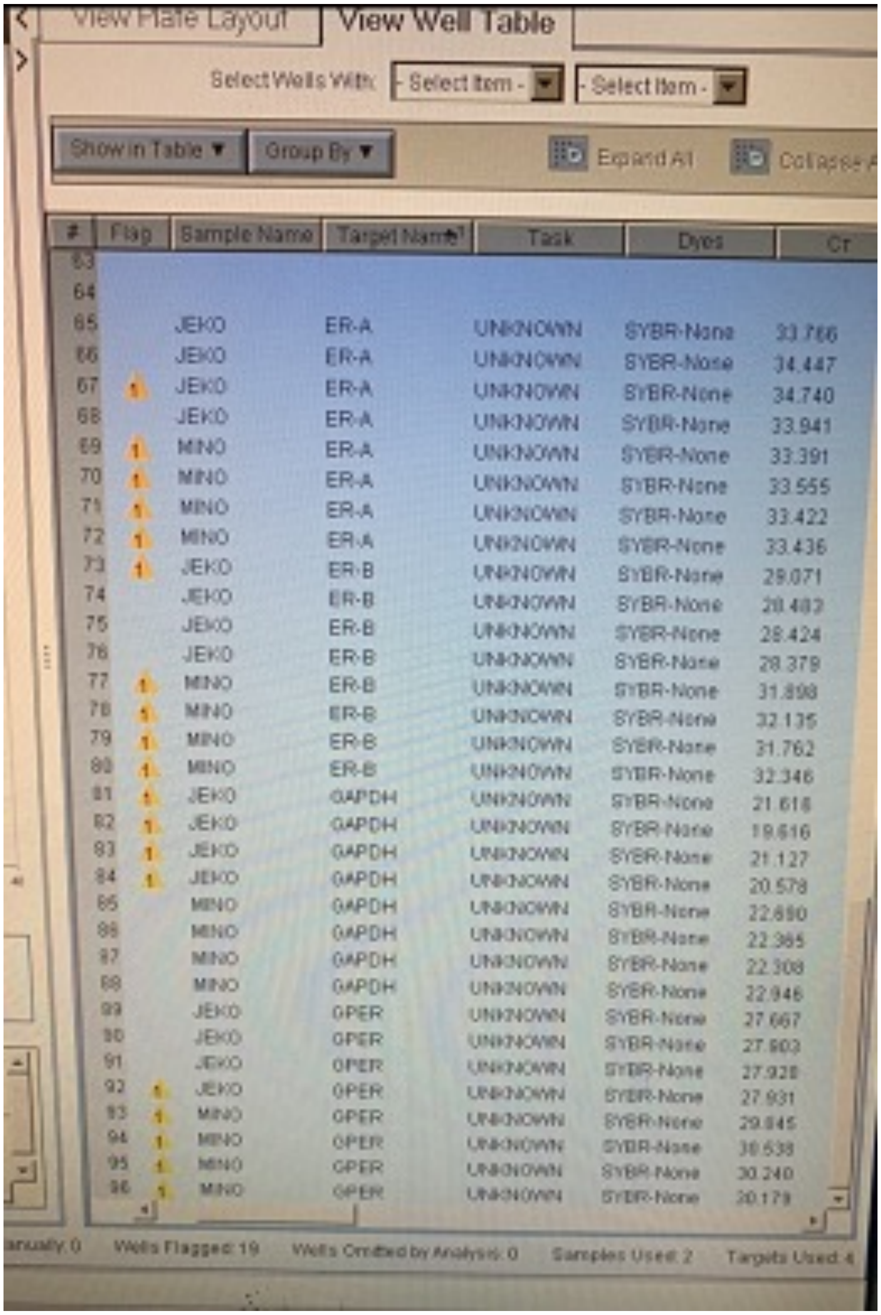

View Plate Layout | View Well Table

Select Wells With: - Select Item - | - Select Item -

Show in Table ▼ | Group By ▼ | Expand All | Collapse A

#	Flag	Sample Name	Target Name	Task	Dyes	Ct
63						
64						
65		JEKO	ER-A	UNKNOWN	SYBR-None	33.766
66		JEKO	ER-A	UNKNOWN	SYBR-None	34.447
67	!	JEKO	ER-A	UNKNOWN	SYBR-None	34.740
68		JEKO	ER-A	UNKNOWN	SYBR-None	33.941
69	!	MINO	ER-A	UNKNOWN	SYBR-None	33.391
70	!	MINO	ER-A	UNKNOWN	SYBR-None	33.555
71	!	MINO	ER-A	UNKNOWN	SYBR-None	33.422
72	!	MINO	ER-A	UNKNOWN	SYBR-None	33.436
73	!	JEKO	ER-B	UNKNOWN	SYBR-None	29.071
74		JEKO	ER-B	UNKNOWN	SYBR-None	28.483
75		JEKO	ER-B	UNKNOWN	SYBR-None	28.424
76		JEKO	ER-B	UNKNOWN	SYBR-None	28.379
77	!	MINO	ER-B	UNKNOWN	SYBR-None	31.898
78	!	MINO	ER-B	UNKNOWN	SYBR-None	32.135
79	!	MINO	ER-B	UNKNOWN	SYBR-None	31.762
80	!	MINO	ER-B	UNKNOWN	SYBR-None	32.346
81	!	JEKO	GAPDH	UNKNOWN	SYBR-None	21.618
82	!	JEKO	GAPDH	UNKNOWN	SYBR-None	19.616
83	!	JEKO	GAPDH	UNKNOWN	SYBR-None	21.127
84	!	JEKO	GAPDH	UNKNOWN	SYBR-None	20.578
85		MINO	GAPDH	UNKNOWN	SYBR-None	22.690
86		MINO	GAPDH	UNKNOWN	SYBR-None	22.365
87		MINO	GAPDH	UNKNOWN	SYBR-None	22.308
88		MINO	GAPDH	UNKNOWN	SYBR-None	22.946
89		JEKO	GPER	UNKNOWN	SYBR-None	27.667
90		JEKO	GPER	UNKNOWN	SYBR-None	27.803
91		JEKO	GPER	UNKNOWN	SYBR-None	27.928
92	!	JEKO	GPER	UNKNOWN	SYBR-None	27.931
93	!	MINO	GPER	UNKNOWN	SYBR-None	29.845
94	!	MINO	GPER	UNKNOWN	SYBR-None	30.538
95	!	MINO	GPER	UNKNOWN	SYBR-None	30.240
96	!	MINO	GPER	UNKNOWN	SYBR-None	30.179

anually: 0 | Wells Flagged: 19 | Wells Omitted by Analysis: 0 | Samples Used: 2 | Targets Used: 4

Sup Figure 2A

	JEKO							
G-36	BLANK	0	0.5	1	2	4	8	10
	0.121	1.032	1.213	1.007	1.005	0.934	1.019	0.985
	0.11	0.975	0.978	1.009	0.907	0.993	0.968	0.934
	0.128	0.885	0.975	0.997	1.005	0.936	0.927	0.897
	MINO							
G-36	BLANK	0	0.5	1	2	4	8	10
	0.043	1.292	1.14	1.229	1.099	1.219	1.132	1.181
	0.085	1.296	1.35	1.444	1.261	1.345	1.36	1.126
	0.043	1.224	1.357	1.108	1.235	1.504	1.373	1.33

Mino

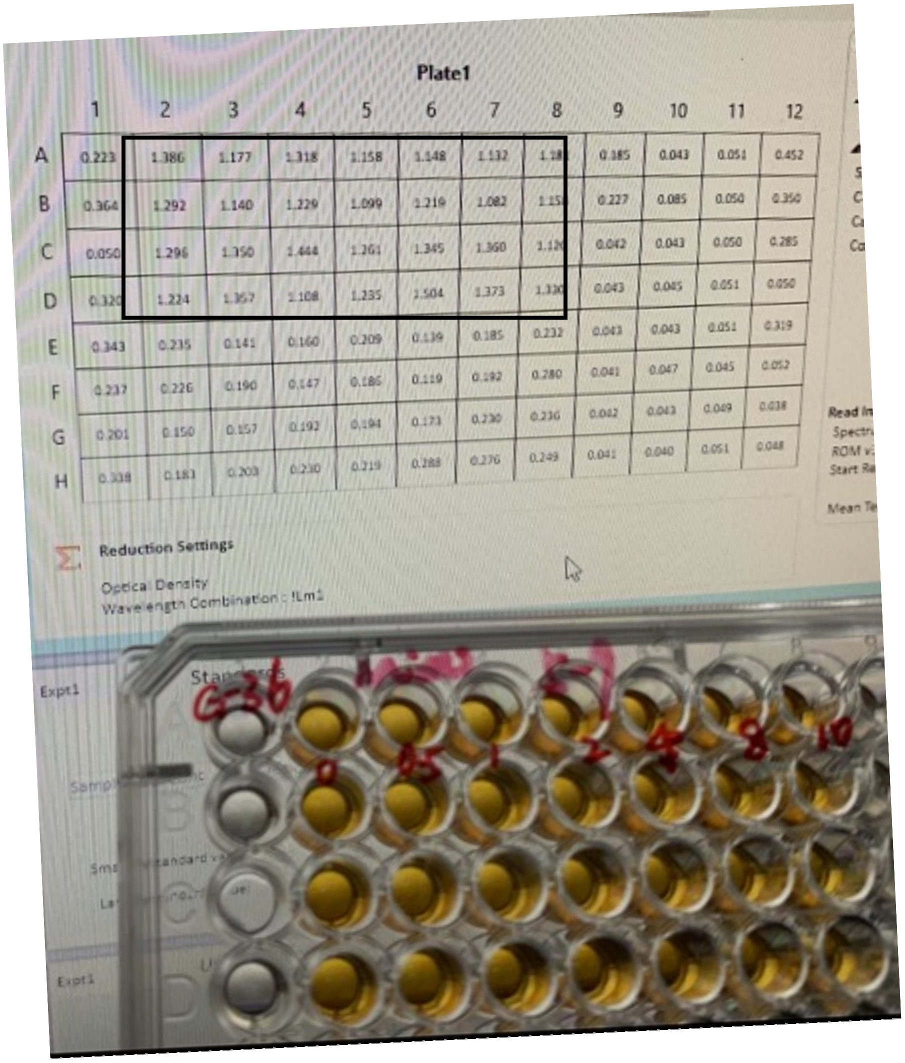

Sup Figure 2B

Jeko-1

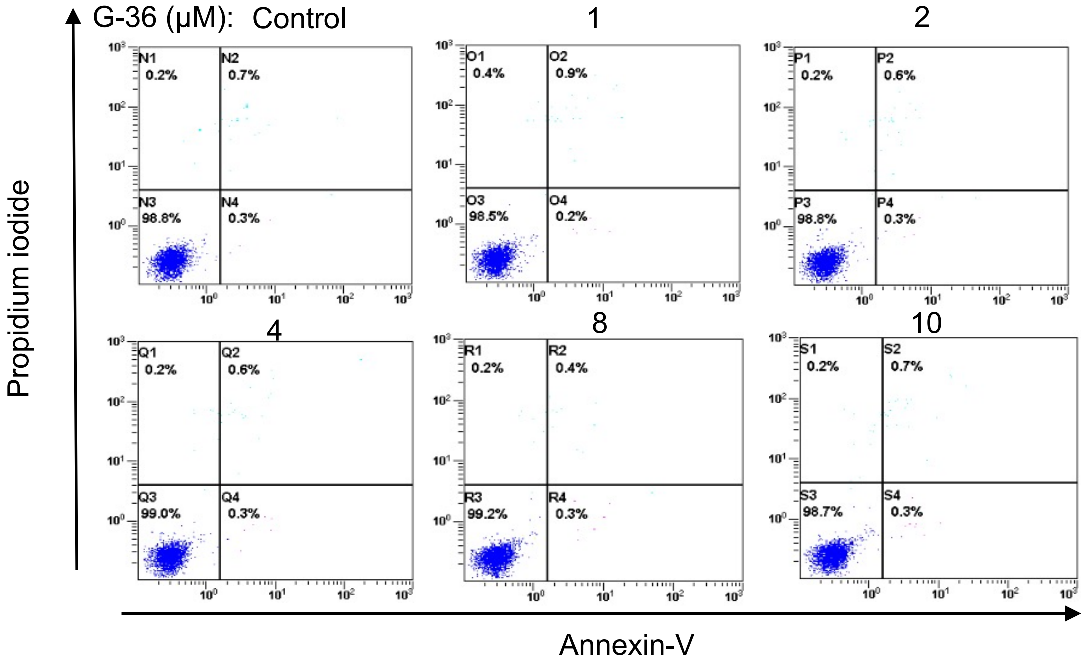

Mino

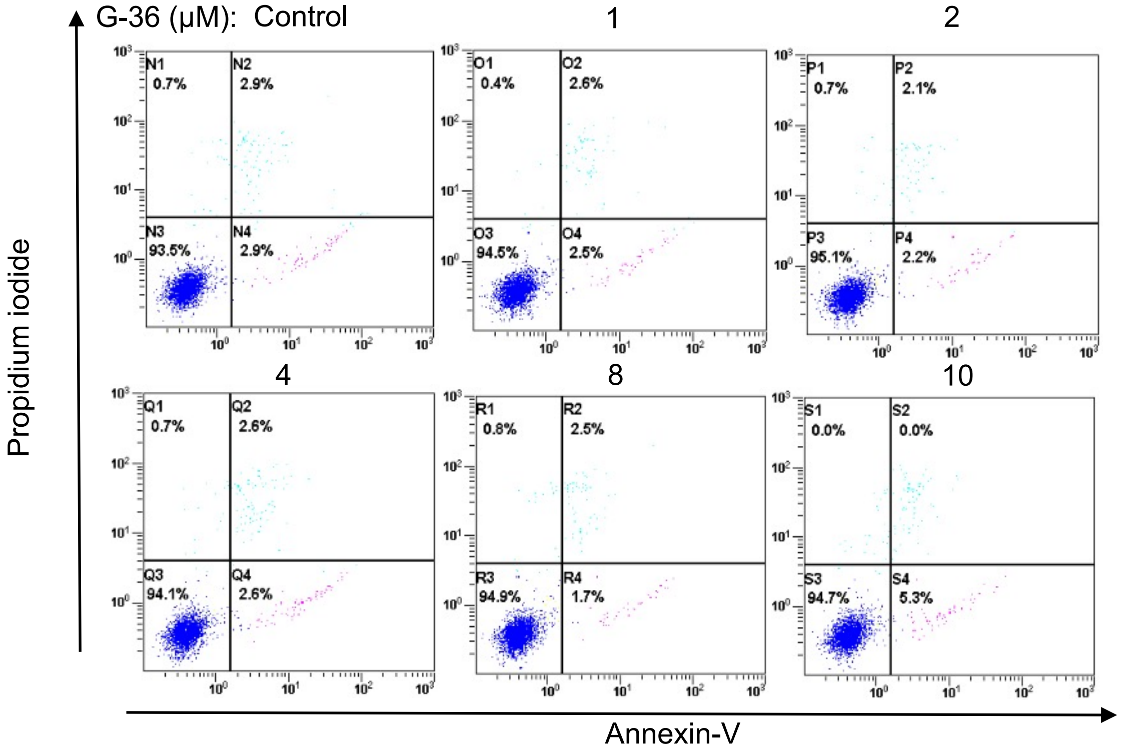

Jeko-1

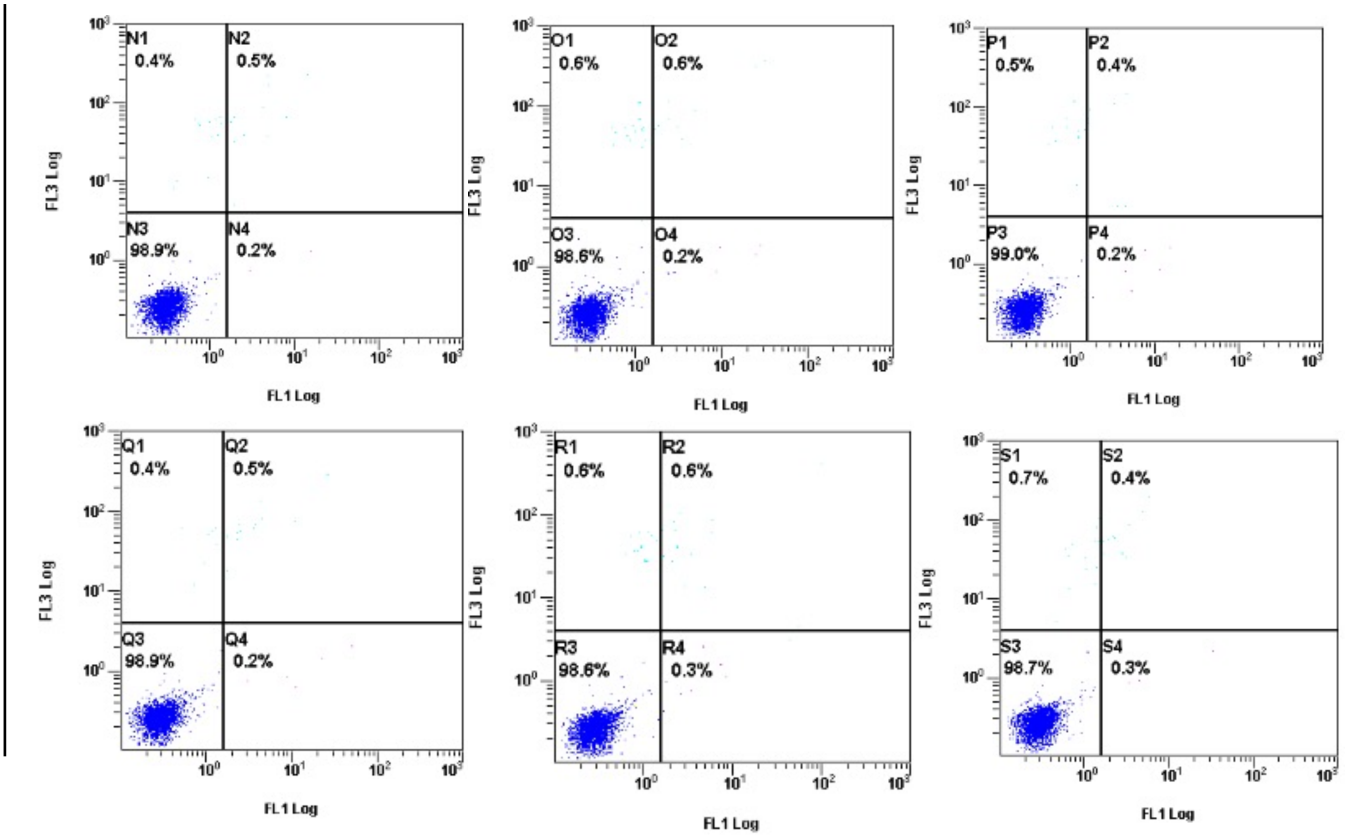
N1 0.4%
N2 0.5%
N3 98.9%
N4 0.2%
O1 0.6%
O2 0.6%
O3 98.6%
O4 0.2%
P1 0.5%
P2 0.4%
P3 99.0%
P4 0.2%
Q1 0.4%
Q2 0.5%
Q3 98.9%
Q4 0.2%
R1 0.6%
R2 0.6%
R3 98.6%
R4 0.3%
S1 0.7%
S2 0.4%
S3 98.7%
S4 0.3%
FL3 Log
FL1 Log

Jeko-1

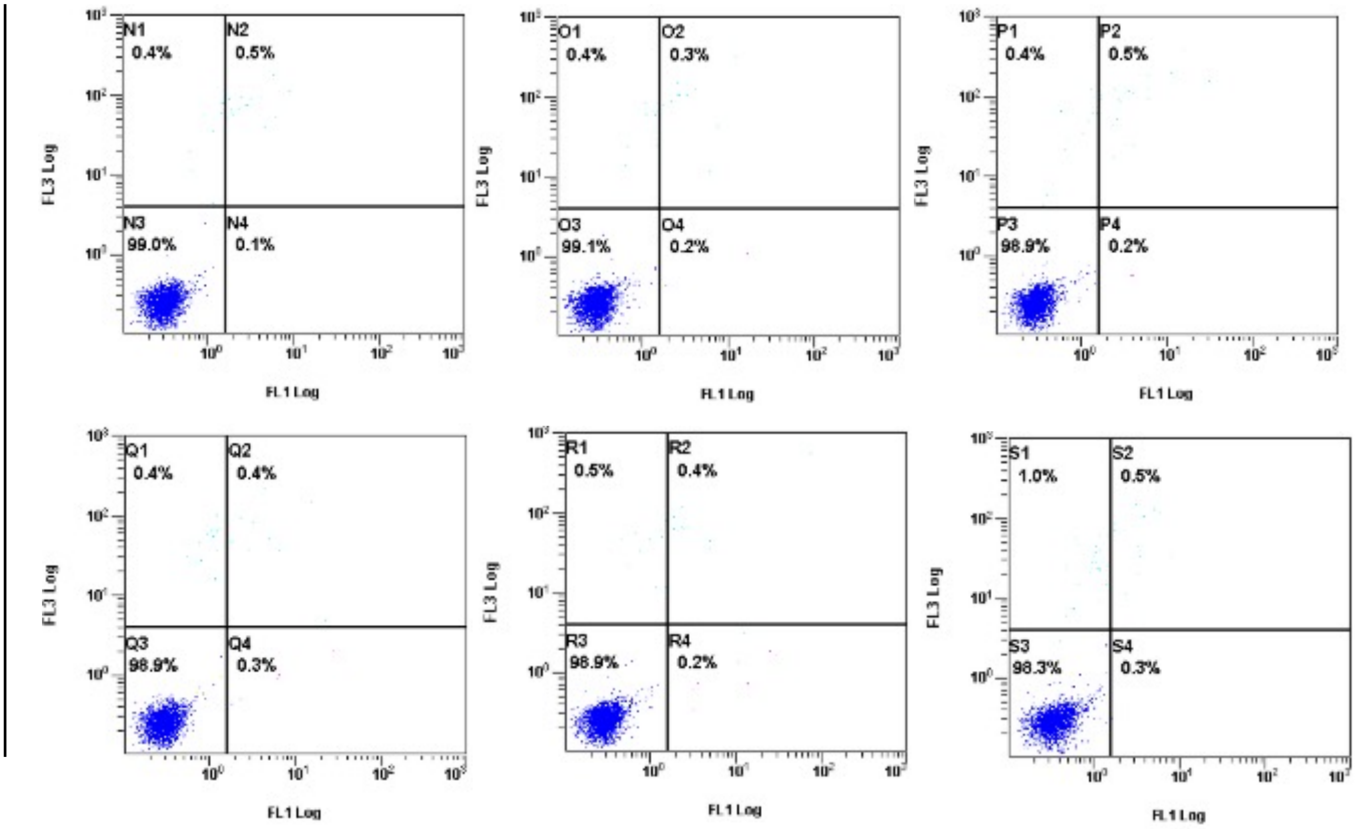
N1 0.4%
N2 0.5%
N3 99.0%
N4 0.1%
O1 0.4%
O2 0.3%
O3 99.1%
O4 0.2%
P1 0.4%
P2 0.5%
P3 98.9%
P4 0.2%
Q1 0.4%
Q2 0.4%
Q3 98.9%
Q4 0.3%
R1 0.5%
R2 0.4%
R3 98.9%
R4 0.2%
S1 1.0%
S2 0.5%
S3 98.3%
S4 0.3%
FL3 Log
FL1 Log

Mino

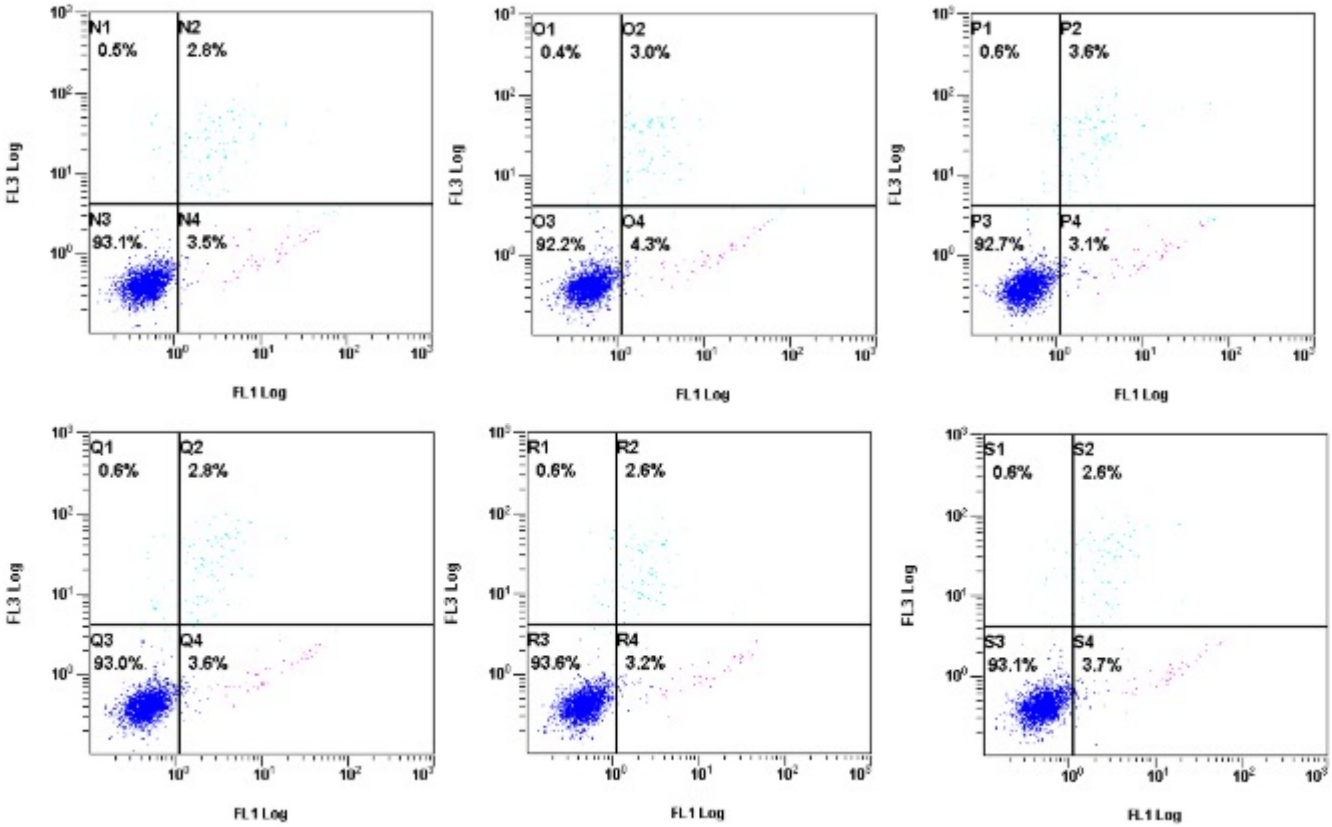
N1 0.5%
N2 2.8%
N3 93.1%
N4 3.5%
O1 0.4%
O2 3.0%
O3 92.2%
O4 4.3%
P1 0.6%
P2 3.6%
P3 92.7%
P4 3.1%
Q1 0.6%
Q2 2.8%
Q3 93.0%
Q4 3.6%
R1 0.6%
R2 2.6%
R3 93.6%
R4 3.2%
S1 0.6%
S2 2.6%
S3 93.1%
S4 3.7%
FL3 Log
FL1 Log

Mino

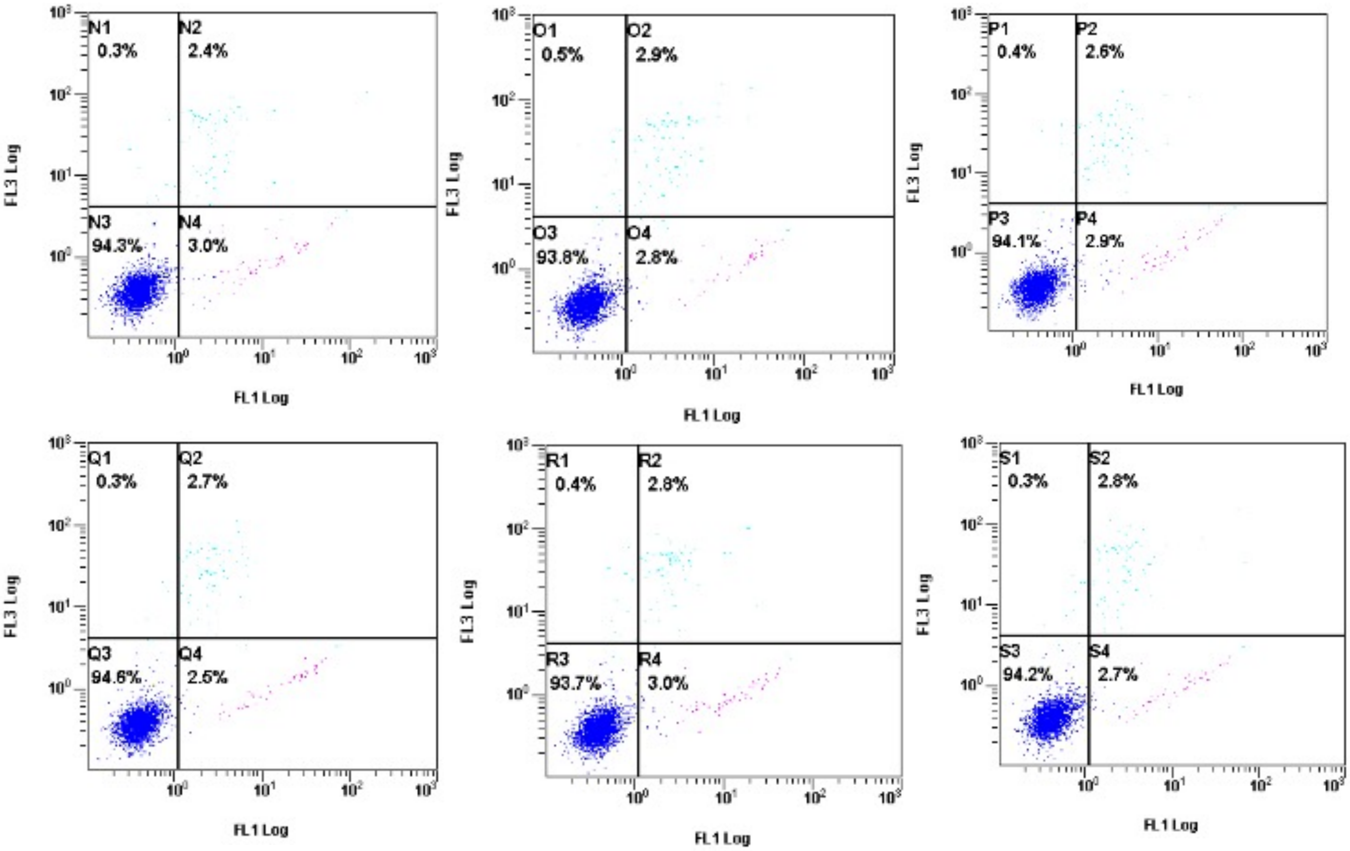
N1 0.3%
N2 2.4%
N3 94.3%
N4 3.0%
O1 0.5%
O2 2.9%
O3 93.8%
O4 2.8%
P1 0.4%
P2 2.6%
P3 94.1%
P4 2.9%
Q1 0.3%
Q2 2.7%
Q3 94.6%
Q4 2.5%
R1 0.4%
R2 2.8%
R3 93.7%
R4 3.0%
S1 0.3%
S2 2.8%
S3 94.2%
S4 2.7%
FL3 Log
FL1 Log

Sup Figure 2C

blank	48h siNC	48h siGPER	96h siNC	96h siGPER
0.102	0.694	0.807	1.403	1.794
0.123	0.793	0.887	1.475	1.762
0.139	0.791	0.845	1.498	1.791

Sup Figure 2D

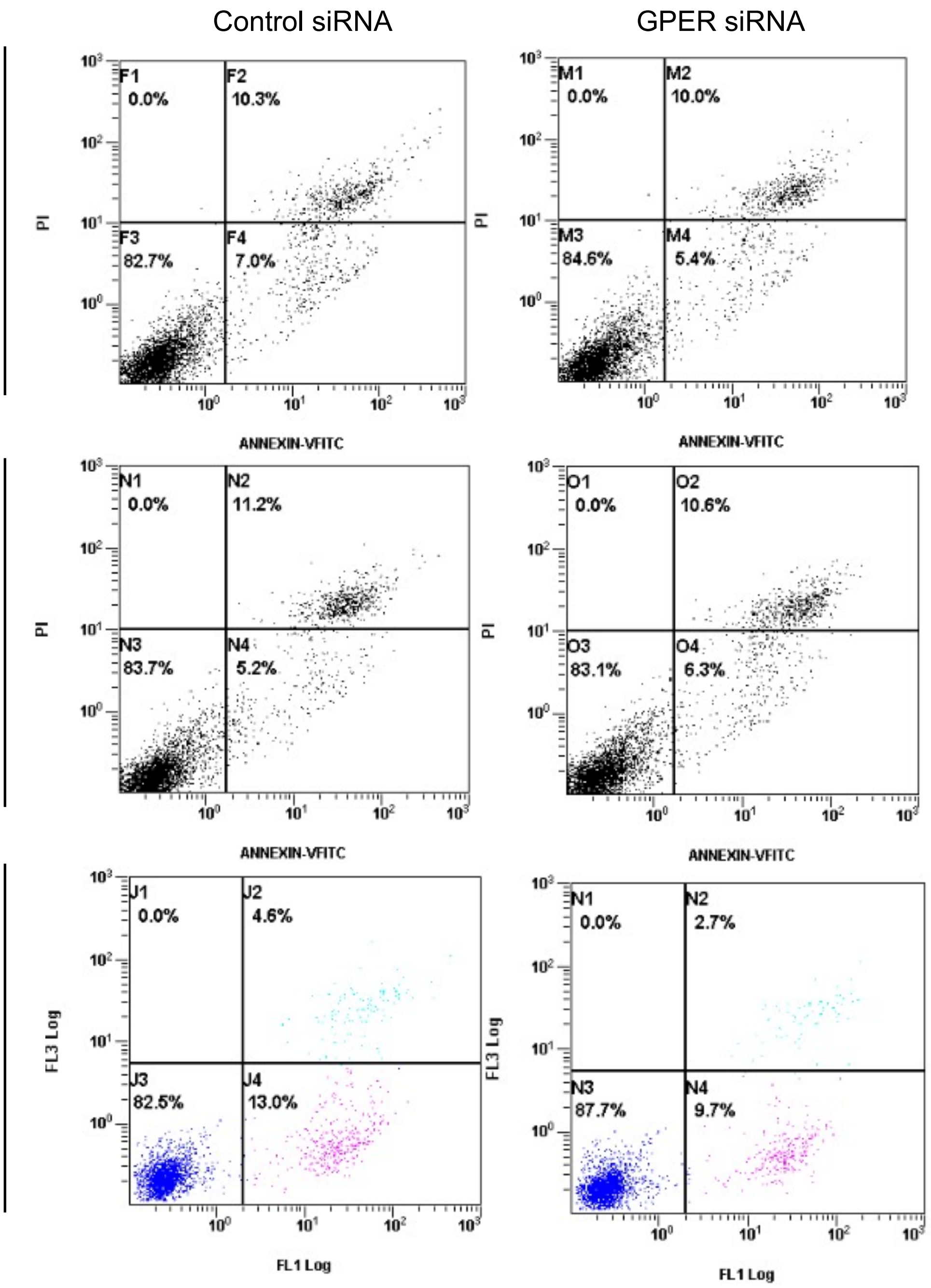

Supplement: Supplementary file 1 [file DataSheet_1.pdf]
