# Supplementary figures and images for: G Protein-Coupled Estrogen Receptor Agonist G-1 Inhibits Mantle Cell Lymphoma Growth in Preclinical Models
Source: Front Oncol. 2021 Jun 15;11:668617. doi: 10.3389/fonc.2021.668617 (PMC8239310; doi:10.3389/fonc.2021.668617)

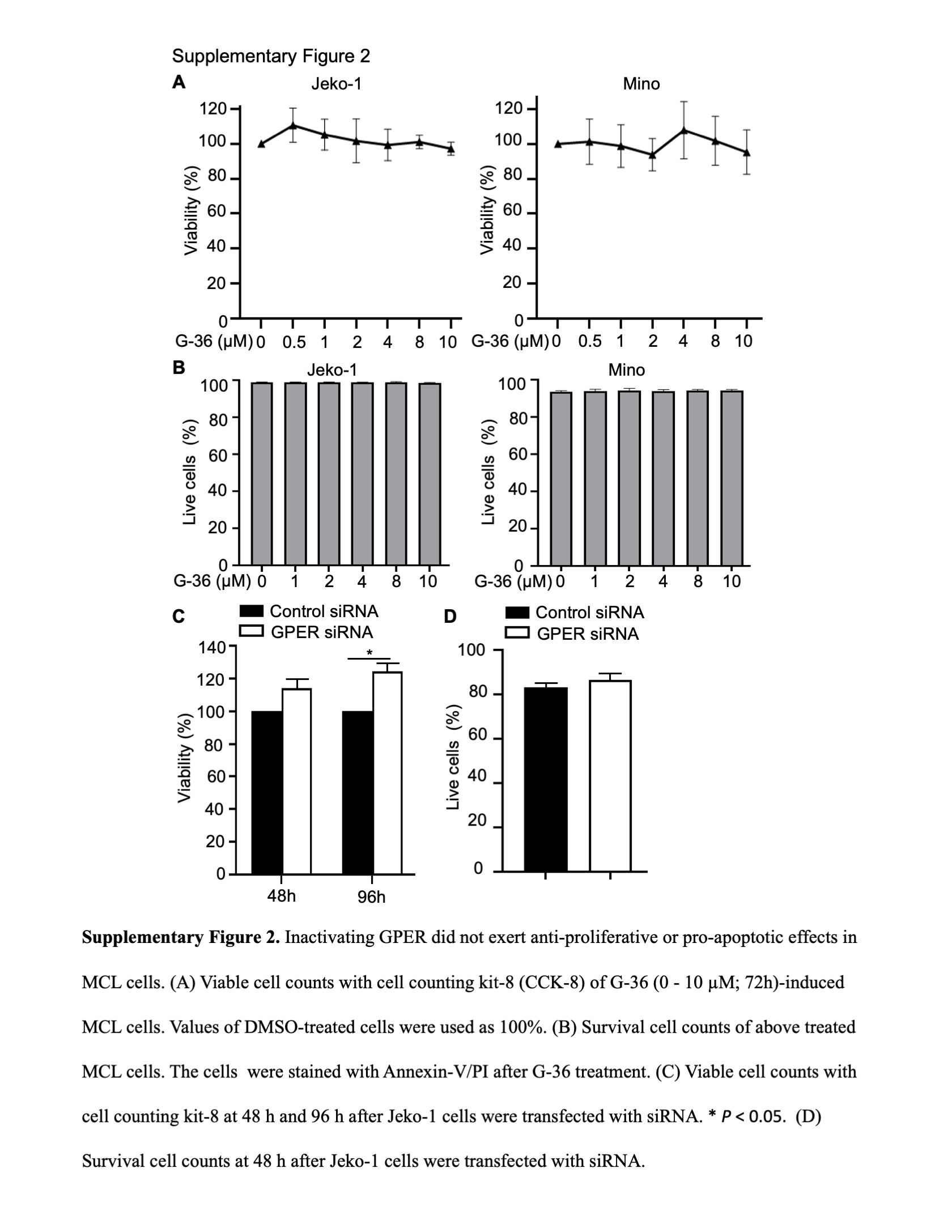

Supplement: Supplementary file 3 [file Image_2.jpeg]
